# Role of murine macrophage in temporal regulation of cortisol- and serotonin-induced adipogenesis in pre-adipocytes when grown together

**DOI:** 10.1242/bio.034629

**Published:** 2018-08-10

**Authors:** Sushri Priyadarshini, Biswaranjan Pradhan, Palok Aich

**Affiliations:** School of Biological Sciences, National Institute of Science Education and Research (NISER), HBNI, PO- Bhimpur-Padanpur, Via- Jatni, District:- Khurda, 752050, Odisha, India

**Keywords:** Microarray, Kinetics, Adipocytes, Macrophages, Co-culture, Psychological stress, Homeostasis, Glucocorticoid, Serotonin

## Abstract

Regulation of adipogenesis, the root cause for obesity, is very poorly understood. However, studies have presented evidence of immuno-metabolic regulation of adipose tissue during periods of chronic psychological stress, leading to adverse conditions related to stress manifestation, including visceral obesity and atherosclerosis. Despite pronounced association of hormonal markers of stress with dys-regulated metabolic states, the contributing signalling events are yet to be established. It is apparent that to understand contributing signalling events we need a model. Although an *in vivo* model is preferred, it is difficult to establish. The current report, therefore, presents an *in vitro* model system for the simulation of adipose tissue in a chronic stress micro-environment by growing pre-adipocytes with macrophages in the presence and absence of stress hormones. In this report, effects of cortisol and serotonin on the kinetics of immune and metabolic changes in adipocytes and macrophage (alone and co-cultured) was studied through whole genome transcriptome profiling. A transition from pro- to anti-inflammatory response in the immune profile of pre-adipocytes, with increasing time in co-culture with macrophages, was observed. This transition was reversed by stress hormones cortisol and/or serotonin.

## Abstract (precis)

**Summary:** Stress-induced obesity is poorly understood *in vivo* at the molecular level. The current report established a novel molecular basis of adipogenesis *in vitro*.

## INTRODUCTION

Adipose tissue, apart from being the primary energy reserve and an important endocrine organ (Kershaw and Flier, 2004), is also a source of fat-derived metabolically active substances (Fain, 2006; Quinkler et al., 2006; Richelsen, 1992; Tang et al., 2015) that are potent pathogenic contributors in modulating systemic inflammatory homeostasis (Fan et al., 2012; Tang et al., 2015). The pathogenic potential of adipose tissue is associated with changes in morphology and cellular functions of the cells that involve (a) aberrant hormonal signalling and (b) excess release of adipokines. Secretion of adipokines leads to systemic low-grade inflammation, as well as altered dynamics of lipid storage to promote secondary diseases through ectopic lipid accumulation, which can lead to metabolic syndromes such as obesity, energy disorders and diabetes (Fan et al., 2012; Suganami et al., 2012; Tang et al., 2015). These disorders, marked by inflammation of adipose tissue, are regulated by infiltration of immune-inflammatory cells and cytokines. Studies have already revealed an increase in the secretion of inflammatory proteins in adipose tissue in obesity (Mraz and Haluzik, 2014; Ohira et al., 2013). The secretion of inflammatory proteins suggests that the resident and infiltrating immune cells in inflamed obese adipose tissue might be an important contributor to inflammation besides the secretion of adipokines. It is also possible that infiltrating immune cells might have also influenced changes in metabolic regulation of adipose tissue (Mraz and Haluzik, 2014; Ohira et al., 2013). The association of adipose and macrophage cells is physiologically important and termed as ATMs (adipose tissue macrophages). ATMs have enormous lipid storage capacity in the form of lipid droplets (Aouadi et al., 2014) and can undergo lipolysis, thereby serving as an important source of free fatty acids and glycerol, which are known regulators of adipose tissue metabolism (Michaud et al., 2013; Saraswathi and Hasty, 2006).

In fact, psychological stress is a well-established contributor to inflammatory metabolic disorders like obesity, diabetes and dyslipidemia (Catalina-Romero et al., 2013; Pouwer et al., 2010; Scott et al., 2012; Tamashiro et al., 2011). Many endocrine factors, such as insulin, catecholamines, insulin like growth factors, growth hormones, glucocorticoids and thyroid hormones influence lipolysis and promote proliferation and differentiation of pre-adipocytes (Chapman et al., 1985; Dicker et al., 2007; Klemm et al., 2001; Lane et al., 1981; Obregon, 2008). Although regulation of adipocyte homeostasis by these endocrine factors has been extensively studied, the signalling events involved are not well understood. Glucocorticoids are well-established hormones, known to be involved in mediating psychological stress responses. Cortisol, a glucocorticoid, is already known to induce adipocyte differentiation as well as promote lipolysis (Feldman and Loose, 1977; Peckett et al., 2011). Research in the past decade has pointed out the peripheral action of serotonin in regulating systemic energy homeostasis, and of serotonin receptors HTR2a and HTR3 in mediating the adipogenic effect of serotonin in adipose tissue (Kinoshita et al., 2010). Serotonin, which is otherwise known as ‘the happy hormone’, is a known neurotransmitter involved in regulating appetite and mood. Enterochromaffin cells in the gut-lining produce 95% of the body's serotonin (Glišić et al., 2006; Young and Leyton, 2002), and it is carried by platelets and mast-cells to the site of inflammation (Dürk et al., 2013; Peckett et al., 2011; Sepiashvili et al., 2013). In cases of chronic psychological stress, stress hormones like cortisol can induce adipocyte hypertrophy (Spesivtseva et al., 1979) and adipose tissue inflammation (Lee and Aich, 2002), making adipose tissue an ideal site for the dumping of serotonin by mast cells and platelets. It has also been reported that adipocytes are themselves capable of producing serotonin and that gut derived serotonin can regulate signalling through HTRs in adipocytes (Stunes et al., 2011; Sumara et al., 2012). So, during a chronic stress episode, inflamed adipose tissue would be subject to very high levels of cortisol and serotonin, and these hormones might together modulate immunological and metabolic functions of inflamed obese adipose tissue. It has been reported that glucocorticoids are capable of up regulating HTRs in adipose tissue (Li et al., 2016), raising speculation that these two hormones might even act together to increase obesity in adipose tissue. In addition to the already known repertoire of genes involved in mediating adipose tissue signalling and function, we hypothesize that there could be a larger network of genes responsive to systemic fluctuations in adipose tissue biology under chronic stress.

In this report, we have explored how stress hormones can modulate immuno-metabolic functions of adipose tissue by co-culturing 3T3L1 pre-adipocyte cells and RAW 247.6 macrophages and treating them with the stress hormones cortisol, serotonin and both cortisol and serotonin together. We have used pre-adipocytes to see how cortisol and serotonin could manipulate naïve cells. The kinetics of the inflammatory and metabolic changes have been followed through whole genome transcriptome profiling at 6, 24 and 48 h for each treatment. The study helped us to identify the systemic changes in pre-adipocytes due to prolonged co-culturing with macrophages, and whether these changes contributed to the adipocyte-macrophage crosstalk. Furthermore, a 48 h time point was chosen to better understand the individual and combinatorial effects of these hormones on co-cultured adipocytes.

## RESULTS

Unless otherwise mentioned, all comparisons were made for pre-adipocytes cultured in the presence of macrophages with respect to time matched pre-adipocytes grown in the absence of macrophages (sometimes referred to as grown alone).

### Kinetics of differentially regulated genes in pre-adipocytes grown in the presence and absence of macrophages

**Fig. 1.**
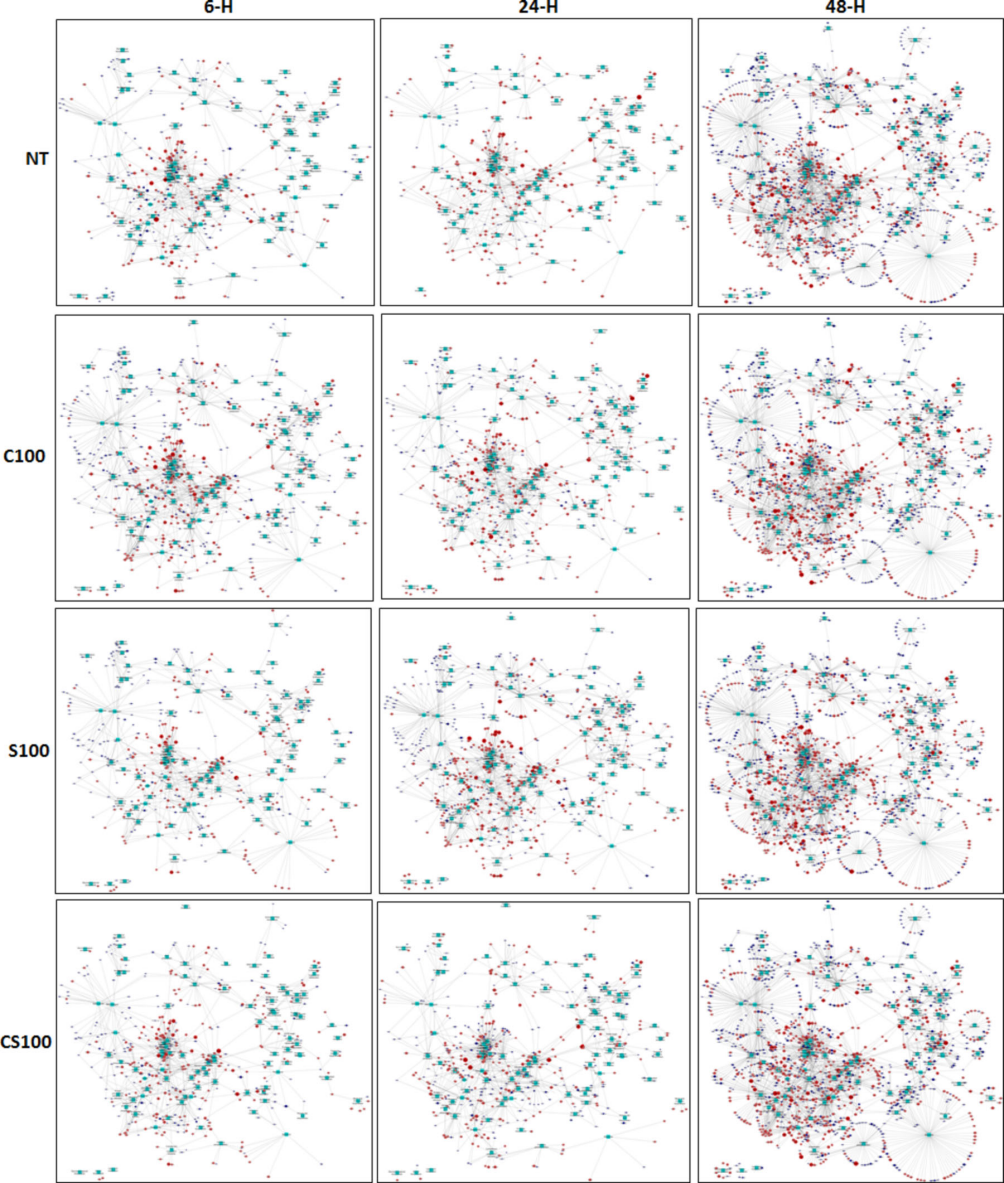
**Gene Network following various treatment conditions.** Gene network significantly enriched pathways and differentially regulated genes in co-cultured adipocytes when compared against adipocytes grown alone at 6 h, 24 h, and 48 h under the following treatment conditions: no treatment, cortisol, serotonin and cortisol-serotonin.

The transcription kinetics of pre-adipocytes co-cultured with macrophages was compared with pre-adipocytes grown alone at 6 h, 24 h and 48 h to identify the genes responsible for interactive modulation of inflammatory and metabolic adipocyte function, and phenotype in co-culture. Fig. 1 shows a qualitative depiction of the kinetic profile of significantly enriched pathways using differentially transcribed genes in each treatment condition at 6 h, 24 h and 48 h (refer to the Supplementary Information for a detailed view and pathway enrichment values). Fig. 1 shows that the total number of differentially regulated genes at the 48 h time point was approximately double that of earlier time-points.

### Comparative inflammatory and metabolic gene expression in pre-adipocytes in the presence and absence of macrophages

**Fig. 2.**
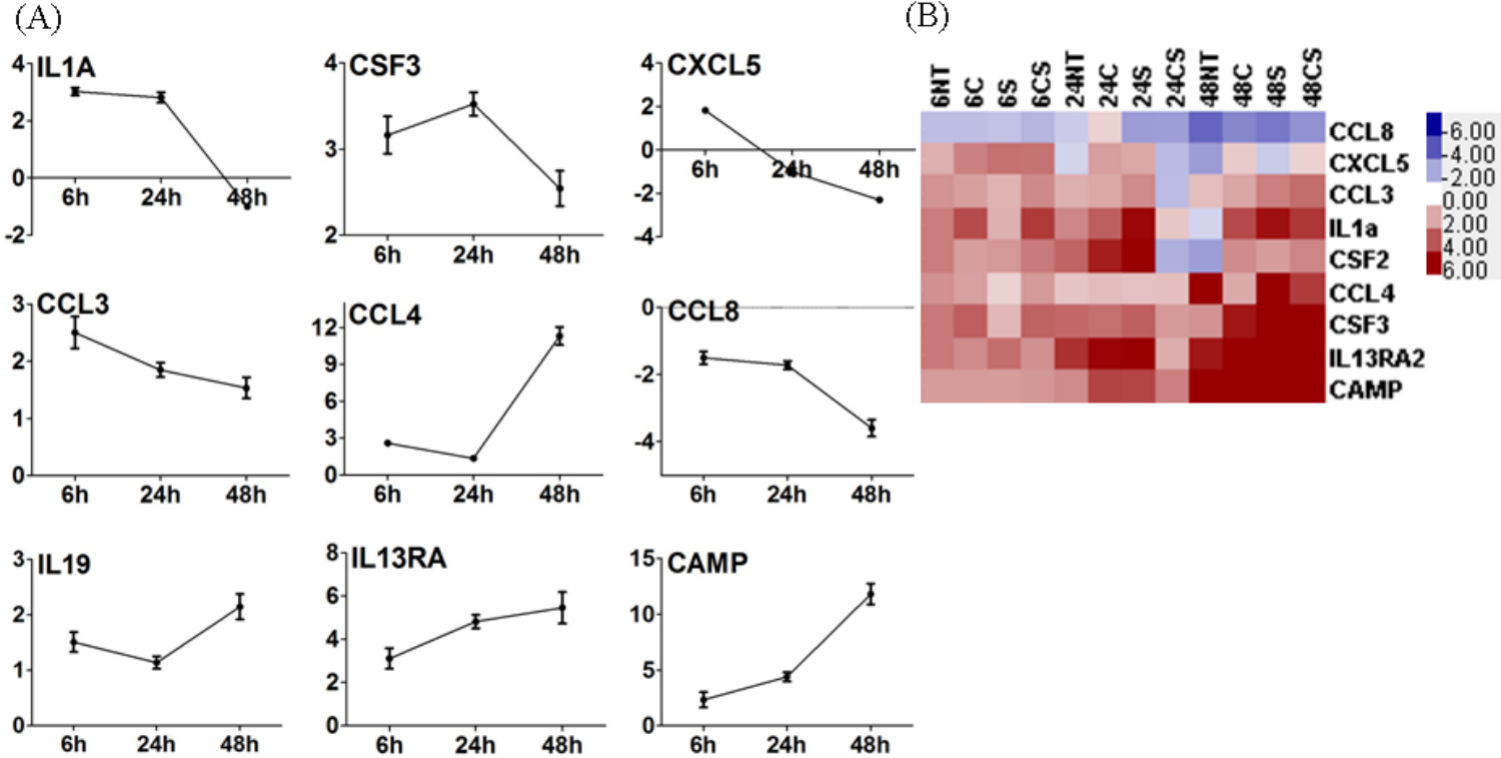
**Time dependent transcriptional changes.** Transcription kinetics of IL1a, CSFSS3, CXCL5, CCL3, CCL4, CCL8, IL13ra, IL19, and CAMP in adipocytes co-cultured with macrophages compared against adipocytes grown alone (A) expressed as fold-changes under no treatment, the fold-changes are plotted as mean±s.d. of the probe replicates in the array (B) expressed as heat-map for fold-changes for no treatment, cortisol, serotonin and cortisol-serotonin treatment conditions.

**Fig. 3.**
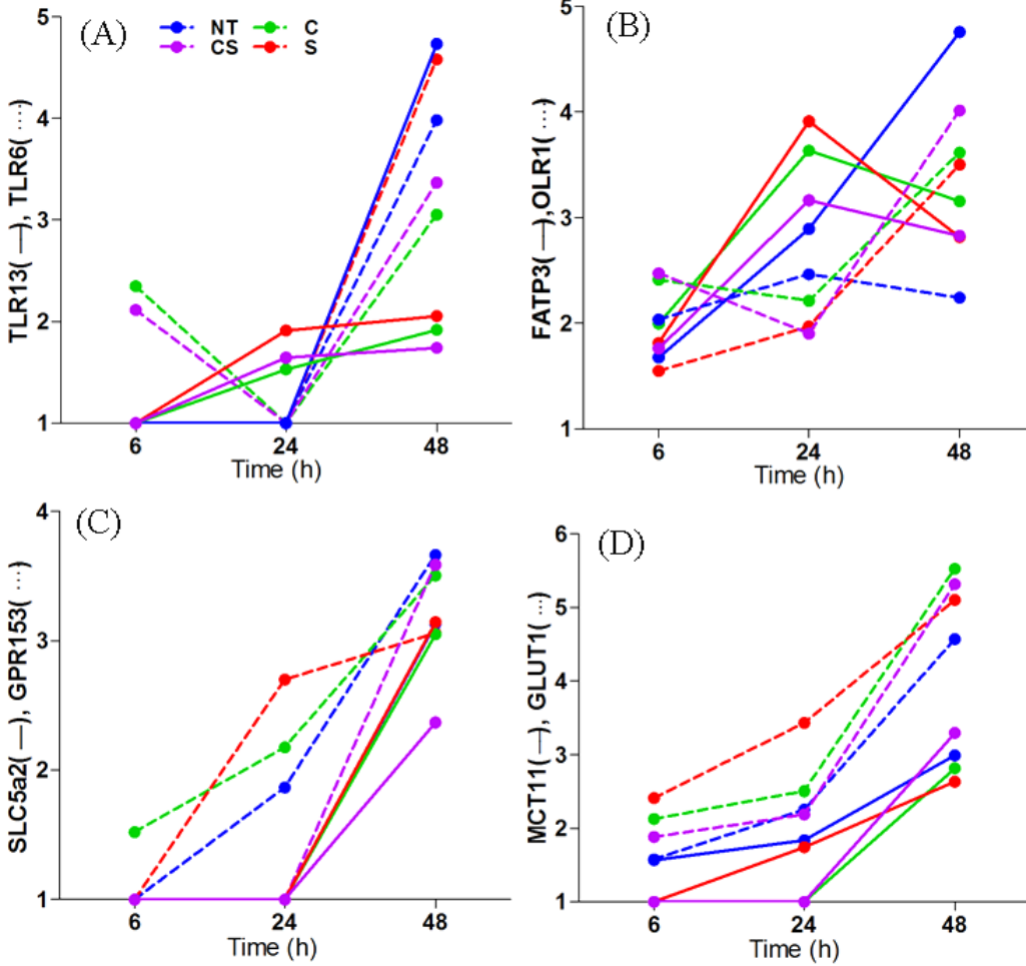
**Transcription kinetics of select genes.** Transcription kinetics at 6 h, 24 h and 48 h of (A) TLR13 and TLR6, (B) OLR1 and FATP3, (C) SLC5a2 and GPR153, and (D) GLUT1 and MCT11 in co-cultured adipocytes compared against adipocytes grown alone and treated for no treatment, cortisol, serotonin and cortisol-serotonin treatment conditions.

**Fig. 4.**
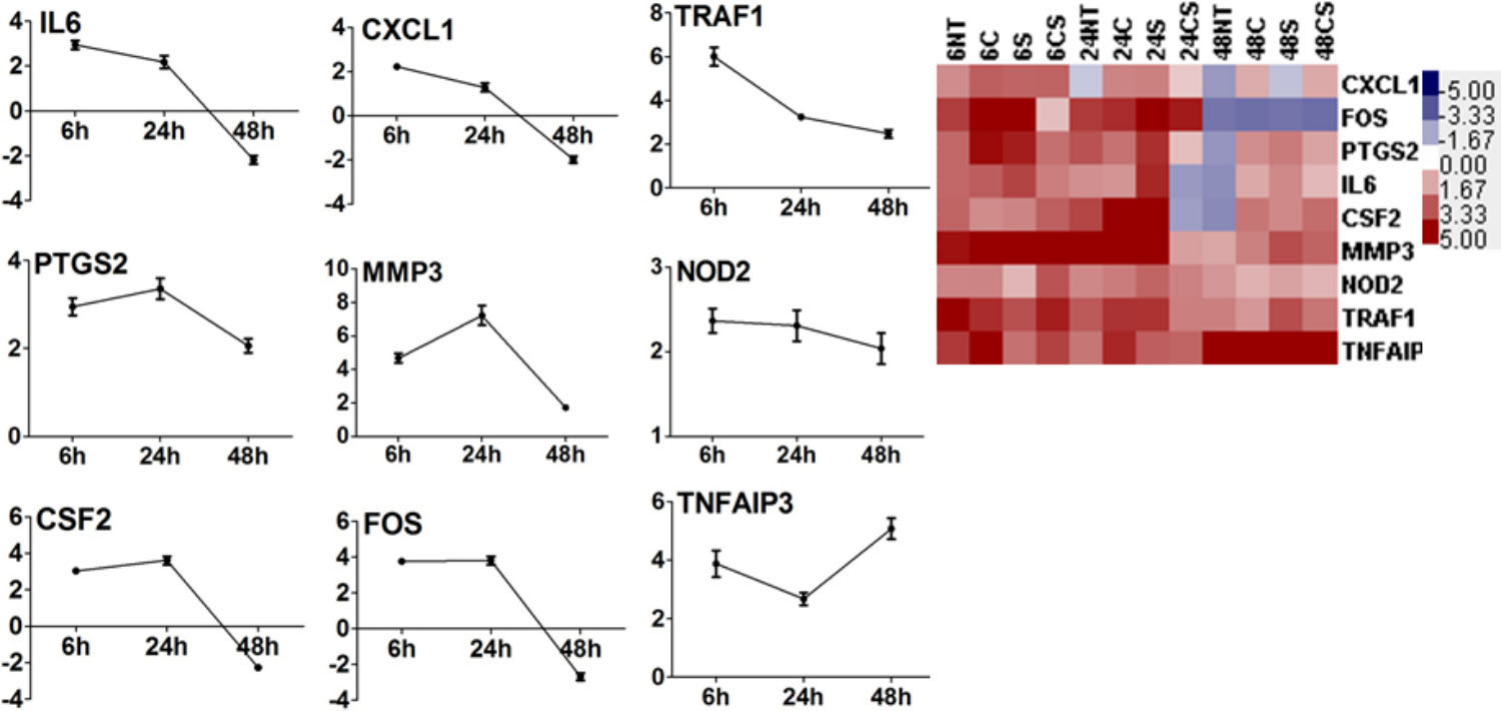
**Time dependent changes in TNF-pathway.** Transcription kinetics at 6 h, 24 h and 48 h of TNF-pathway down-stream genes: IL6, CXCL1, TRAF1, PTGS2, MMP3, NOD2, TNFαip3, CSF2, FOS in adipocytes co-cultured with macrophages compared against adipocytes grown alone (A) expressed as fold-changes under no treatment, the fold-changes are plotted as mean±s.d. of the replicates of probes in the array (B) expressed as heat-map of fold-changes for no treatment, cortisol, serotonin and cortisol-serotonin treatment conditions.

We studied the changes in expression of several inflammatory cytokines and chemokines (e.g. IL1A, CSF3, CXCL5, CCL3, CCL4, CCL8, IL19, IL13RA and Camp) in the pre-adipocytes, as a result of co-culturing with macrophages with respect to time matched pre-adipocytes without macrophages. Under no treatment condition IL19, cAMP and CCL4 showed increased transcription with time, however, transcription of IL1A, CSF3, CXCL5, CCL3, CCL4, CCL8 and IL13RA either remained constant or dropped at 48 h (Fig. 2). Transcription of CSF3 increased with increasing time due to treatment with cortisol, serotonin or cortisol-serotonin, although its transcription appeared to decrease with time under the no treatment condition. Cortisol, however, appeared to suppress pro-inflammatory CCL4 while increased expression of anti-inflammatory IL13Ra and cAMP with time until 48 h (Fig. 2). Among the inflammatory pathways that were significantly affected was the NF-кB and TLR signalling pathway. It was observed that transcription of MIP1α and MIP1β, two known chemotactic agents for macrophages, also increased with time in co-cultured adipocytes, with maximum transcriptional expression at 48 h. This increase in transcription of MIPs was almost always accompanied by an increase in the transcriptional expression of TLR2/6 complex (Fig. S1). Unlike TLR6, TLR2 transcription appeared to decrease with time due to co-culturing, and serotonin appeared to cause higher transcription of TLR6 compared to cortisol at 48 h (Fig. 3A). At 48 h, TLR13 (a newer endosomal receptor that recognizes bacterial 23S rRNA) too showed a more than fourfold increase in transcription under the no treatment condition, while treatment with cortisol, serotonin and cortisol-serotonin reduced the transcription of this gene twofold. NF-кB signalling pathway showed a gradual increase in activity with increasing time, with maximum activity at 48 h. Although TLR2/6 mediated NF-кB pathway activation is known to induce apoptosis, our data showed that viability of the cells (as determined through MTT assay) was uncompromised even after 48 h treatment. In fact, the apoptosis pathway was suppressed at 48 h (Fig. S2). Many of these inflammatory proteins are known to be responsive to activation of activator protein 1 (AP-1), which is a transcriptional regulator composed of members of the Fos and Jun families of DNA binding proteins (Zenz et al., 2008). FOS, an early response transcription factor, showed maximum transcription at 6 h, which decreased gradually with time for all conditions. Both cortisol and serotonin enhanced its transcription from an approximately fourfold increase (no treatment) to 11-fold and sixfold, respectively, at 6 h (Table 1). Similarly, JUN also showed increased transcription when treated with cortisol and serotonin (Autelitano, 1994), however, the changes were not as pronounced as FOS (Table 1). Increase in the lipid content of co-cultured adipocytes led us to believe that co-culturing either increased the importing of fatty acids into adipocytes or induced *de novo* lipid synthesis in them. Analysis of the transcription profile of lipid transporters revealed that transcription of both OLR1 and FATP3 increased steadily with time. Cortisol and serotonin treatment upregulated OLR1 transcription and FATP3 transcription appeared to be suppressed by treatment with cortisol-serotonin (Fig. 3B). We noticed that SLC5a transcription increased sharply after 24 h for all the treatment conditions (Fig. 3C). Certain groups of G-protein receptors are known to bind to short chain fatty acids. GPR153, one of the genes of the same GPCR family, also showed a constant increase in transcription with time due to co-culturing (Fig. 3C). Like SLC5a, transcription of this gene did not vary with different treatments. Cytoplasmic acetyl-coenzyme A synthetase 2 (ACSS2) was also upregulated and its transcription increased with time. Besides, ACSL1 (long-chain fatty acid, CoA ligase 1), responsible for the conversion of free long-chain fatty acids into fatty acyl-CoA esters, was suppressed at 48 h. However, ACSL4 that preferentially converts arachidonic acid (AA) to arachidonate was upregulated at 48 h. The conversion to arachidonate that is responsible for lipid import into the cells, as well as those required for intra-cellular compartmentalization, was affected. Apart from fatty acid transport, genes that could possibly contribute to *de novo* fat synthesis also increased due to co-culturing. GLUT1 transcription increased more than fourfold at 48 h, and although both cortisol and serotonin increased the transcription of this gene, there was no significant difference between cortisol, serotonin and cortisol-serotonin treatments (Fig. 3D). Increased transcription of GLUT1 was accompanied by a less pronounced, yet significant increase in the transcription of MCT11, a mono-carboxylate transporter (Fig. 3D). This suggested that co-culturing favoured higher adipogenicity in these cells through lipid and glucose import, although this does not solely account for the entire metabolic regulation in these cells. It was also observed that most of the downstream TNF signalling pathway genes, such as IL6, CXCL1, PTGS2, MMP3, CSF2, FOS, NOD2, TRAF1, TNFΑIP3, showed differential expression patterns with time. Under the no treatment condition, the transcription of the majority of these genes decreased at 48 h, except for TNFαip3, which showed increased expression at 48 h (Fig. 4). The temporal transcription pattern of these genes remained similar to the no treatment condition following treatment with cortisol, serotonin and cortisol-serotonin, although each of these treatments increased the transcription of these genes compared to the no treatment condition (Fig. 4). Comparative transcriptional analysis of genes selected as representative of pro-inflammation and metabolic profiling suggested that macrophage induced adipogenic and pro-inflammatory responses.

**Table 1.**
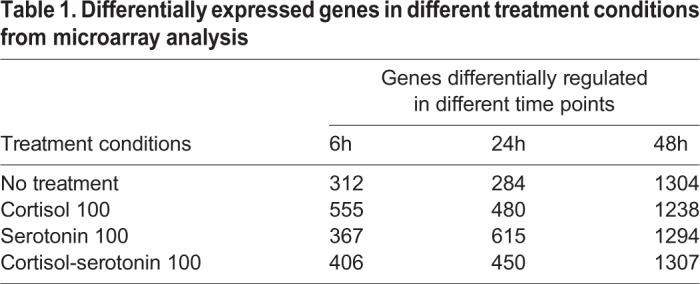
**Differentially expressed genes in different treatment conditions from microarray analysis**

### Role of macrophage in TNF and associated signalling to induce adipogenesis in pre-adipocytes

**Fig. 5.**
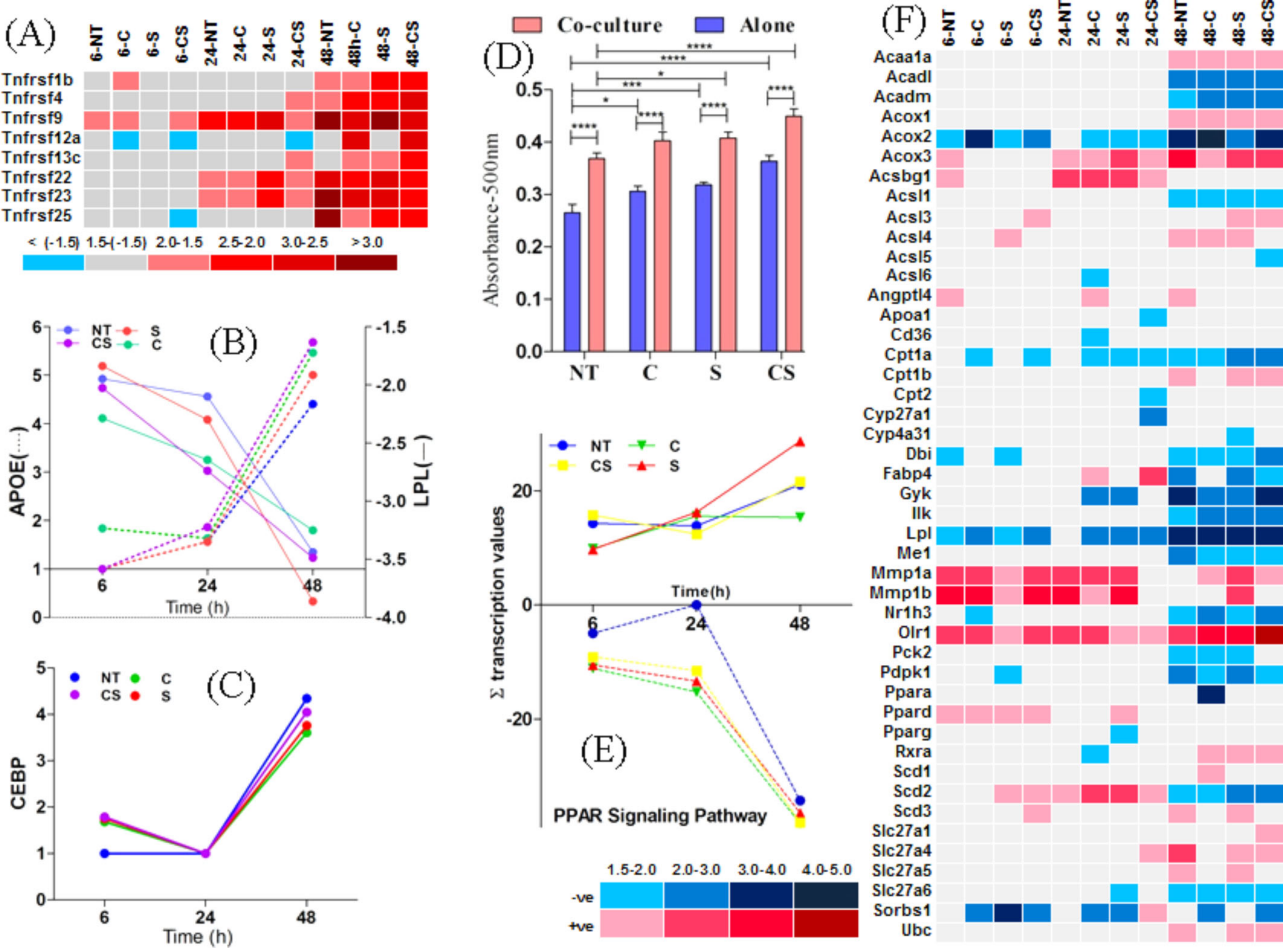
**Time dependent transcriptional profile of TNF receptors.** Transcription kinetics of (A) TNF receptors expressed as a heat-map of fold-changes with a cut-off of±1.5. (B) APOE and LPL (C) CEBP in adipocytes co-cultured with macrophages compared against adipocytes grown alone under no treatment, cortisol, serotonin and cortisol-serotonin conditions. (D) Oil Red O absorbance of co-cultured adipocytes and adipocytes grown alone treated with no treatment, cortisol, serotonin and cortisol-serotonin at 48 h; absorbance values are shown as mean±s.d. and significant differences are indicated by asterisks *P<0.05, **P<0.01, ***P<0.001 and ****P<0.0001. (E) Summation of differential transcription values of all genes of PPAR signalling pathway in co-cultured adipocytes compared against adipocytes grown alone at 6 h, 24 h and 48 h for each treatment condition. (D) Heat-map of fold-changes of the differentially expressed genes in PPAR signalling pathway with a cut-off value of±1.5, at 6 h, 24 h and 48 h for each treatment condition.

Studies have associated inflamed obese tissue with higher secretion of TNFα, and its role in regulating adipocyte metabolism has already been reported (Maachi et al., 2004). Our analysis revealed that TNFα did not show significant variation with time but TNF soluble receptors, known to initiate the PI3K-AKT signalling pathway, showed a significant increase at 48 h (Fig. 5A) in co-cultured adipocytes. TNFα signalling is already known to decrease transcription of lipoprotein lipase (LPL) (Cawthorn and Sethi, 2008) which is the rate-limiting enzyme for hydrolysis of triglycerides in triglyceride rich proteins. Further analysis revealed that in co-cultured adipocytes, LPL transcription decreased with time for all the treatments (Fig. 5B). It was also noted that CEBP transcription was less than twofold until 24 h, however, the transcription increased to fourfold at 48 h for all treatment conditions (Fig. 5C). The decrease in LPL transcription was accompanied by increasing accumulation of lipid droplets in co-cultured adipocytes (Fig. 5D). Like CEBP, APOE transcription increased to four- to fivefold at 48 h (Fig. 5B), which correlated with the drop in TNF-signalling at 48 h. APOE transcription is already known to be affected by PPARγ (Yue and Mazzone, 2009). The data analysis revealed that PPARγ transcription did not vary significantly due to co-culturing. In fact PDPK1, which activates PPARγ and promotes adipocyte differentiation (Yin et al., 2006), was downregulated at 48 h (Fig. 5D). The PPAR signalling pathway appeared to be affected after 24 h concomitant with the decrease in TNF-signalling (Fig. 5E). It was observed that transcription of stearoyl-CoA desaturase 2 (SCD2), known to reduce adiposity, increased until 24 h followed by a drop at 48 h. Transcription of acyl-CoA dehydrogenase genes (both long-chain ACADL and medium-chain ACADM) was suppressed at 48 h, while CPT1a decreased over time, indicating that mitochondrial beta-oxidation of fatty acids did not occur at 48 h. Meanwhile, enzymes for peroxisomal oxidation of fatty acids like acetyl-CoA C-acyltransferase 1 (ACAA1) A, ACOX1 and ACOX3, which help in unsaturation of long chain fatty acids, were upregulated. Transcription of Acsbg1 (acyl-CoA synthetase), responsible for activation and beta-oxidation of very long-chain fatty acids (Steinberg et al., 2000), increased until 24 h, followed by decrease at 48 h (Fig. 5F). Glycerogenesis causes re-esterification of fatty acids, thus restraining the release of free fatty acids. PPARγ/RXRα complex is required for activation of PCK2 (PEPCK2) (Devine et al., 1999; Tontonoz et al., 1995) – which in turn is required for glycerogenesis in adipocytes for re-esterification of fatty acids to triglycerides – and was also down-regulated at 48 h. Results revealed the molecular basis of lipidogenesis induced by the macrophage in pre-adipocytes.

### Effects of cortisol and serotonin on pre-adipocytes and macrophages

**Fig. 6.**
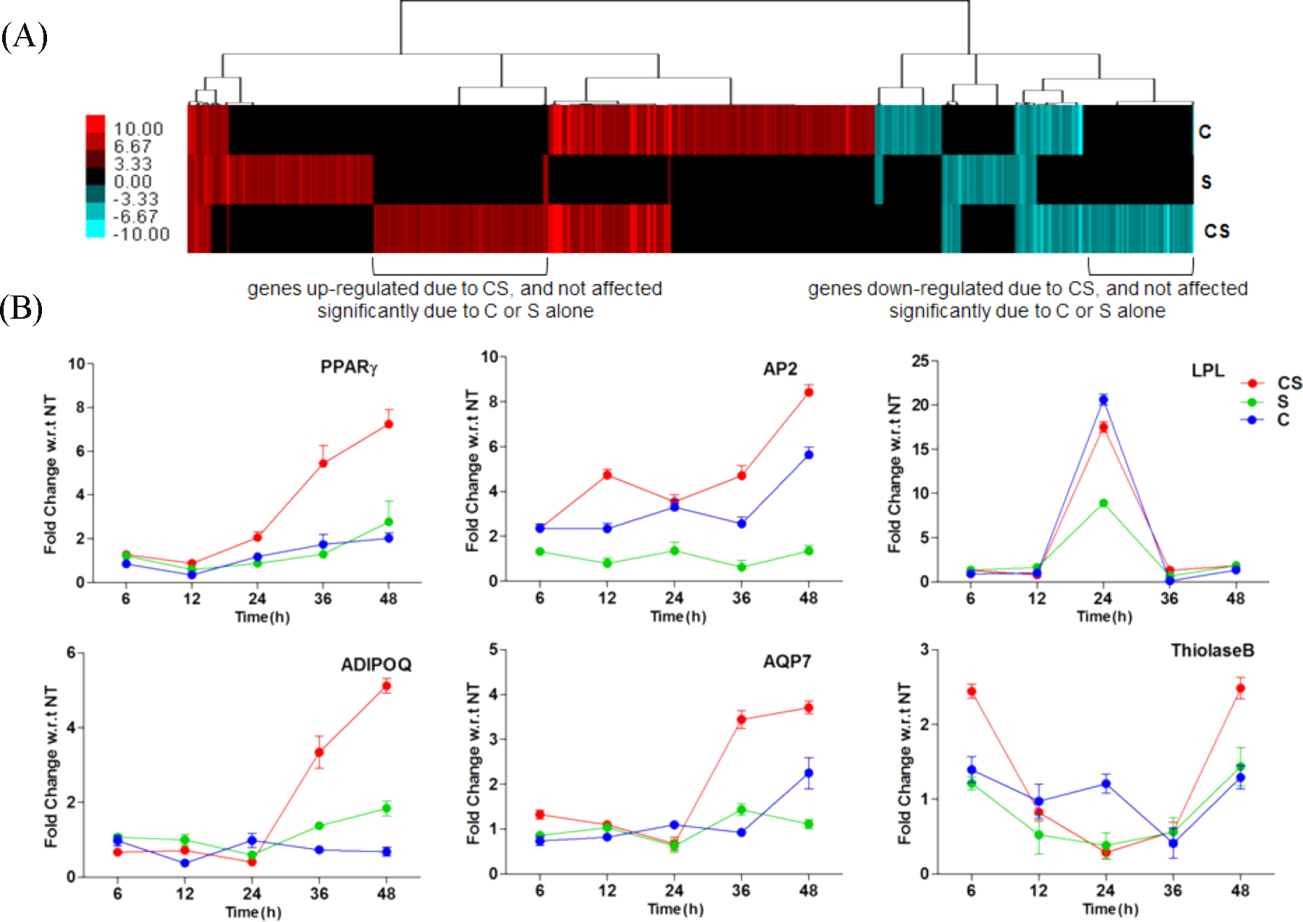
**Kinetic profile of adipogenesis genes.** (A) Differentially expressed genes in co-cultured adipocytes treated with cortisol, serotonin and cortisol-serotonin compared against no treatment expressed as a heat-map of fold-changes with a cut-off of± 2.0. (B) RT-PCR determined relative transcription of PPARγ, AP2, LPL, ADIPOQ, AQP7 and Thiolase B in co-cultured adipocytes treated with cortisol, serotonin and cortisol-serotonin expressed as fold-changes against time-matched untreated controls at 6 h, 12 h, 24 h, 36 h, and 48 h. Relative fold changes are shown as mean±s.d.

The results so far establish the role of macrophages in enhancing adipogenesis in pre-adipocytes. We wanted to further establish the role of the stress associated hormone cortisol in the presence and absence of serotonin. This is particularly important as our results revealed that cortisol regulated certain serotonin receptors in pre-adipocytes to perhaps induce adipogenesis. We identified 167 genes that did not show significant differential expression until 24 h, however their transcription increased from 2- to 18-fold at 48 h in pre-adipocytes that were co-cultured with macrophages (Table S1 contains a list of these genes, a minimum cut-off of a twofold change was used to identify these genes). Results revealed that at 48 h a major shift in transcriptional activation occurred. To further explore the metabolic and immune regulatory changes in adipocytes co-cultured with macrophages at 48 h, a whole genome transcriptional study was carried out to see the effect of stress hormones on co-cultured adipocytes, in which co-cultured adipocytes each treated independently with either cortisol, or serotonin, or cortisol-serotonin, were compared with untreated co-cultured pre-adipocytes. It was found that certain group of genes were differentially expressed with twofold or higher changes (with respect to untreated pre-adipocytes) due to treatment with cortisol-serotonin only; these genes remained suppressed when treated individually with cortisol or serotonin. Fig. 6A shows the differential expression of these genes as a heat-map. Table S2A shows the genes that were up- or downregulated by serotonin, but remained suppressed due to either cortisol or cortisol-serotonin, and Table S2B shows genes that were up- or downregulated by cortisol; however, both serotonin and cortisol-serotonin treatment suppressed them. It was also observed that AA metabolism was affected in cortisol-serotonin as well as serotonin treated co-cultured pre-adipocytes, however, it was not affected due to cortisol treatment. The fatty acid elongation pathway was also affected due to serotonin. However, no contextually relevant pathways were sufficiently populated with high transcription values due to these treatments (the details of enriched pathways due to treatment with cortisol, serotonin and cortisol-serotonin can be found in Table S3).

**Fig. 7.**
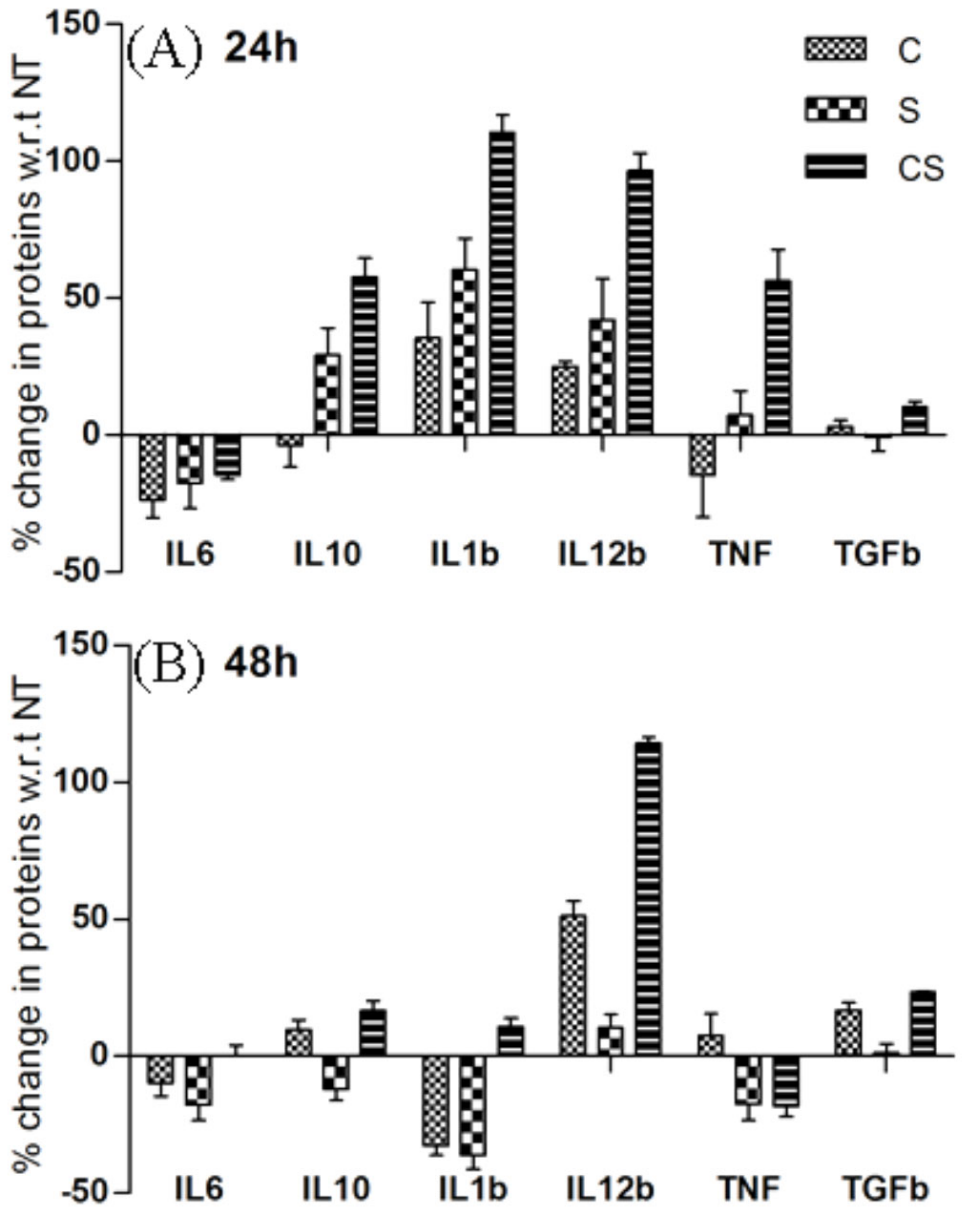
**Inflammatory responses in adipocytes.** Percentage changes of IL6, IL1b, IL10, IL12b, TNF, TGFb at (A) 24 h and (B) 48 h in co-cultured adipocytes treated with cortisol, serotonin and cortisol-serotonin compared against untreated time-matched controls as determined through ELISA are expressed as mean±s.d. and significant differences are indicated by asterisks *P<0.05, **P<0.01, ***P<0.001 and ****P<0.0001.

Although treatment with cortisol-serotonin increased the lipid content of co-cultured pre-adipocytes compared to no treatment (Fig. 5D), microarray analysis did not show a significant change in PPARγ transcription in cortisol, serotonin, and cortisol-serotonin treated co-cultured pre-adipocytes. qRT-PCR based transcriptional profiling of PPARγ and AP2 across multiple time-points, however, revealed that transcription of both the genes increased over time in co-cultured pre-adipocytes, and treatment with cortisol-serotonin caused higher transcription than cortisol or serotonin treatment individually. It was also observed that transcription of AQP7 (aquaporin) and ADIPOQ (adiponectin) increased after 24 h and treatment with cortisol-serotonin caused the highest transcription of these four genes. Cortisol-serotonin treatment caused maximum transcription of Thiolase B at 6 h, which gradually decreased with time until 36 h, and again increased at 48 h. Cortisol or serotonin treatment, however, did not significantly affect the transcription value of any of these genes (except AP2, which was upregulated due to cortisol treatment at 48 h) more than twofold compared to non-treated pre-adipocytes. LPL showed no increase in transcription due to treatment with cortisol, serotonin or cortisol-serotonin at any time-point, except at 24 h where its transcription was very high (Fig. 6B). Current results revealed that adipogenesis is further accentuated in pre-adipocytes grown together with macrophages in the presence of cortisol-serotonin and adipogenesis is greater compared to pre-adipocytes grown in the absence of macrophages, but irrespective of the presence of cortisol, or serotonin or cortisol-serotonin (Fig. 5D). Similarly, we observed the percentage increase of anti-inflammatory (IL10 and TGFβ) and pro-inflammatory genes (IL1b, IL6, IL12 and TNF) in macrophages, grown alone as well as co-cultured with pre-adipocytes, through ELISA. It was seen that both the pro- and anti-inflammatory cytokines were expressed in cortisol, serotonin and cortisol-serotonin treated macrophages grown alone as well as co-cultured (Fig. 7). It was also observed that the secretion of these inflammatory cytokines decreased with time. This result is particularly important in order to establish if there are any changes in immune activity of macrophages in the presence of pre-adipocytes as well in the presence of cortisol, serotonin or cortisol-serotonin.

## DISCUSSION

Adipose tissue contains a resident population of immune cells (in particular macrophages and lymphocytes), along with pre-adipocytes. Previous studies have demonstrated that chronic overnutrition leads to increased infiltration of macrophages in obese adipose tissue, resulting in pre-adipocyte hypertrophy (Suganami and Ogawa, 2010; Sun et al., 2011), accumulation of lipids in macrophages and dysregulated pro-inflammatory and anti-inflammatory cytokine content (Surmi and Hasty, 2008). In this report, genome wide microarray analysis revealed that even when adipocytes and macrophages are not in direct contact with each other, co-culturing causes transcription activation of large number of genes in pre-adipocytes, and the number of genes increase significantly over a period of 48 h (as shown in Fig. 1). Recent studies have indicated that during energy surplus pre-adipocyte hypertrophy is associated with reduced pre-adipocyte turnover (Arner et al., 2010) and may promote inflammation induction of hypoxia and aberrant extracellular matrix remodelling (Pasarica et al., 2009). It was found that metalloproteinases, like MMP10 and MMP3 that have already been implicated in extra cellular tissue remodelling in adipocytes (O'Hara et al., 2009), were transcribed at a higher rate in adipocytes due to co-culturing. Higher expression of metalloproteins suggests that crosstalk between adipocytes and macrophages in adipose tissue contributes to adipose tissue expansion and remodelling (Sanada et al., 2013) and there might be large number of paracrine factors that exert inflammatory and metabolic regulation in obese adipose tissue.

### Both *de-novo* lipid synthesis and triglyceride acquisition contribute to increased lipid content in co-cultured pre-adipocytes

It was seen that co-cultured pre-adipocytes had a higher lipid content than pre-adipocytes grown alone. The high lipid content of co-cultured pre-adipocytes could be directly attributed, in part, to increased activity of genes like CEBP, APOE and OLR1 that have established roles in adipogenesis, and partly to a decrease in LPL expression. During lipid excess triglycerides become associated with apolipoproteins. Triglyceride rich lipoproteins become enriched in APOE and are lipolyzed on the surface of endothelial cells by LPL that mediates release of fatty acids from circulating lipoproteins. It is established that adipocytes are also capable of producing APOE (Zechner et al., 1991), which is capable of modulating adipocyte lipid and lipoprotein metabolism. Earlier studies show a correlation between the increase in APOE mRNA and the cellular lipid content, which corroborates our findings. It is also reported that LPL shows early expression which gradually decreases with time, while APOE is late expressing and increases with time (Zechner et al., 1991), which is also supported by the results of the current report. Apart from the actions of CEBP and APOE, it appears that pre-adipocyte–macrophage co-cultures are rich in extra-cellular oxidized lipids, which are directly transported into the adipocytes, as is evident by the higher expression of the OLR1 gene in co-cultured adipocytes. Higher expression of APOE and OLR1 in cortisol-serotonin treated pre-adipocytes also correlates with the higher lipid content of these cells (Fig. 5).

There are also other mechanisms that could contribute to adipogenesis, e.g. *de novo* synthesis of fatty acids from the cytoplasmic pool of acetyl-CoA. The transport of glucose into co-cultured pre-adipocytes increases with time as evidenced by the increase in GLUT1 expression. Glucose, after being converted to acetyl CoA, can either undergo oxidation through mitochondrial TCA cycle, or can be transported through ATP citrate lyase to the cytoplasm where it is converted to malonyl-CoA for subsequent long-chain fatty acid synthesis. ACCA1 (acetyl-CoA carboxylase 1), which catalyses conversion of acetyl-CoA to mal-CoA, is upregulated, while carnitine palmitoyltransferase 1 (CPT-1) is downregulated at 48 h, implying that import and oxidation of long chain fatty-acids in mitochondria might be blocked. This might contribute to the increased triglyceride synthesis in co-cultured pre-adipocytes at 48 h. It is also established that the expression of SLC16A11 (MCT11) is capable of altering lipid metabolism, most notably causing an increase in intracellular triacylglycerol levels (Williams et al., 2014). MCT11 expression also peaked at 48 h in co-cultured pre-adipocytes, implying that mono-carboxylate transporters mediated pyruvate import, which contributed to the increased lipid content of co-cultured pre-adipocytes.

### Role of adipogenic mediators in inflammation

Once inside the cytoplasm, the selective fatty acids are carried to different subcellular locations by fatty acid binding proteins (FATPs) for fatty acid synthesis or oxidation. Although FATP3 was upregulated due to co-culturing, acyl-CoA dehydrogenases and CPT1 (responsible for transport and oxidation of long-chain fatty acids in mitochondria) were suppressed, suggesting that long-chain fatty acids are transported into co-cultured pre-adipocytes and are targeted to peroxisome (which has a preference for very long-chain fatty acids) rather than mitochondria for oxidation. Differential regulation of ACSL1, ACSL3 and ACSL4 in co-cultured pre-adipocytes at 48 h further indicated that different types of fatty acids are present in the pre-adipocytes. Suppression of ACSL1 indicated that although there is fatty acid intake, long-chain fatty acids are not activated to esters, thereby blocking their metabolism. Upregulation of ACSL4, known to have a preference for transport of AA, and its conversion to arachidonate indicates active AA metabolism in co-cultured pre-adipocytes. This is further supported by the upregulation of arachidonate metabolizing enzyme PTGES. Possible ω-hydroxylation of AA and LTB_4_ by the CYP4f group (see Table S1) of enzymes further supports the fact that AA metabolism is enhanced at 48 h in co-cultured pre-adipocytes. Stress hormones appear to downregulate FATP3 at 48 h, implying that cortisol and serotonin probably contribute to higher lipid accumulation, either by dampening the oxidative degradation of long-chain fatty acids or by reducing the transport of long-chain fatty acids into sub-cellular compartments like mitochondria or peroxisomes. Prostaglandins, by-products of arachidonate metabolism, bind to prostanoid receptor G-protein-coupled receptors, leading to increased cyclic AMP concentrations and the activation of a number of signalling transduction cascades. The decrease in PTGS2 (COX2) expression over time (Fig. 2A) hints that prolonged co-culturing might induce an AA-metabolism mediated anti-inflammatory state in pre-adipocytes. Stress hormones like cortisol and serotonin cause higher expression of COX2 (Fig. 2B) and therefore reinforce a pro-inflammatory state in co-cultured pre-adipocytes. Recently it has been suggested that short-chain fatty acids can bind to certain group of G-protein-coupled receptors and trigger inflammation in adipocytes. In this report, upregulation of GPR153 (a member of the same family) at 48 h parallel with increasing adipogenicity, and decreasing pro-inflammation pins down the potential role of short-chain fatty acids as well as this receptor in mediating inflammation. Apart from the production of lipid oxidation by-products, an increase in GLUT1 transcription also presents the possibility of high glucose oxidation. This can result in production of reactive oxygen species, which are a potent inflammation causing agent and might also contribute to the inflammatory status of co-cultured pre-adipocytes.

### Crosstalk between pre-adipocytes and macrophages causes a shift from pro-inflammatory to anti-inflammatory status over time

Our results indicate that although co-cultured pre-adipocytes secrete more pro-inflammatory proteins (IL1a, IL6, CXCL1, PTGS2, CXCL5, CSF2, CSF3, CCL3, CCL4 and MMP3), their secretion decreases with time spent in co-culture. The decrease in the pro-inflammatory transcription profile was accompanied by an increasing secretion of anti-inflammatory proteins like IL19 (a member of the anti-inflammatory IL10 family), IL13Ra and cAMP during co-culture (Fig. 2). It appears that co-culturing pre-adipocytes with macrophages induces a shift from a pro-inflammation to an anti-inflammation secretory profile. This was also supported by the decreasing transcription of FOS, NOD2 and TRAF1 with time, which we attribute to co-culturing (Fig. 4). FOS and JUN dimerize to form AP1 transcription factor, which is essential for activation of a large number of pro-inflammatory cytokines. Similarly, NOD2 is also among the DNA-binding proteins that are required for transcription activation of inflammatory proteins. However, in the presence of the stress hormone cortisol, it appeared that there was a decrease in pro-inflammation and an increase in chemotraction in immune cells, as supported by higher expression of CSF2, CSF2, CSF3 and CXCL5/ RANTES. Serotonin appeared to contribute more to adipose tissue remodelling through MMP3, but also contributed to pro-inflammation, which decreased with time (Fig. 2). This suggested that most probably in hypertrophied adipocytes, cortisol and serotonin act to aggravate the local inflammation by attracting macrophages and extra-cellular matrix remodelling.

Previous studies have reported a link between increased levels of TNFα with macrophage infiltration into adipose tissue (Cawthorn and Sethi, 2008; Fujiya et al., 2014; Weisberg et al., 2003). Our results also support this observation and showed significant activation of TNF signalling pathway in pre-adipocytes due to co-culturing. Although TNF-signalling pathway appeared to be activated due to co-culturing, the signalling, however, decreases with time, as supported by an increase in the production of TNFΑip3 parallel to the drop in the transcription of pro-inflammatory proteins. TNFαpi3 (A20) is a negative feedback regulator of TNF-signalling pathway and NF-κB, and plays essential roles in the homeostasis by preventing inflammation and apoptosis. Both cortisol and serotonin appear to boost secretion of TNFαip3 in pre-adipocytes and thus prevent adipocyte death due to heightened inflammation. This also corroborates well with the anti-inflammatory boosting of cAMP and IL13Ra by the stress hormone cortisol. TNFα and IL6 respond to immune and metabolic changes and can be produced by activation of TLR-signalling through AP1 transcription factor, or through NF-κB. Although TLR signalling appears to contribute to the inflammatory profile of co-cultured pre-adipocytes (Fig. S1) through TRAF6-mediated TLR2/TLR6 complex, the comparative transcriptional change in TLR2 was not significant. The role of TLR13 has not yet been well explored, however it appears to be involved in the crosstalk between pre-adipocytes and macrophages, and interestingly is suppressed by stress hormones. This suggests a potential role for TLR13 in immune-metabolic regulation. It is also interesting to note that, excluding FOS, the inflammatory gene transcription profile of the 24 h cortisol-serotonin treatment was similar to the 48 h no treatment group, indicating that cortisol and serotonin, when acting together, might bring the system close to homeostasis under prolonged activity.

### Inflammatory molecules as sensors of metabolic status

TNFα production is responsive to both immunological and inflammatory regulators and can orchestrate lipid mobilization from pre-adipocytes by inhibiting LPL, CEBP and PPARγ. Although PPARα, γ or δ are not affected due to co-culturing, activation of PPAR signalling pathway after 24 h shows that PPAR signalling genes could possibly be regulated by other factors, and there could be other PPARγ-independent mechanisms of adipogenesis in co-cultured pre-adipocytes. Increased expression of adipogenic mediators after 24 h was almost parallel to the increase in expression of TNF soluble receptors in co-cultured pre-adipocytes after 24 h; however, an accompanying significant TNFα expression was absent. This result implies that TNF signalling in co-cultured pre-adipocytes is essentially paracrine in nature. TNF is established to regulate LPL transcription by downregulating it (Bulló et al., 2002; Kern, 1997). Studies have shown that in isolated adipocytes TNF did not inhibit LPL (Kern, 1988) but did inhibit LPL in whole adipose tissue pieces (Fried and Zechner, 1989), suggesting its role as a paracrine factor. Many other studies have shown that macrophages are the major source of TNFα in adipocyte–macrophage co-cultures (Suganami et al., 2005). In this case too, co-cultured pre-adipocytes show TNF pathway activation without significant TNF mRNA transcription in pre-adipocytes, strongly suggesting that the TNFα is paracrine in nature and has to come from the macrophages. As has been discussed already, co-culturing induces arachidonate metabolism through COX2, which decreases with time along with TNF signalling. The resulting accumulation of arachidonate-metabolism by-products like prostaglandins that are known to induce pro-inflammation thus also decrease with time. Numerous studies have established that macrophages are capable of producing arachidonate (Teslenko et al., 1997), which can be taken up by co-cultured pre-adipocytes (Lu et al., 2004). Thus, co-cultured macrophages could also act as a possible alternate source of arachidonate that can cause chronic energy overload in pre-adipocytes accompanied by pro-inflammatory decline in a paracrine manner. Similarly, expression of IL4Ra also increased at 48 h in co-cultured pre-adipocytes, without an accompanying expression of IL4 (Table S1). Although IL4 has not been established as a potent paracrine regulator of metabolic activity in pre-adipocytes, our report identifies IL4 as a candidate inflammatory cytokine that could be possibly produced by co-cultured macrophages, that could act in a paracrine loop in adipocytes and contribute to metabolic regulations. It has also been seen that adipose tissue TNFα content increased with increasing obesity, however, extremely obese people had relatively low TNFα levels (Kern, 1997). This implies that TNF signalling is controlled by a very strong feedback regulatory mechanism, as is also suggested by the decrease in TNF signalling with increasing adipogenesis in our report. Since the decrease in TNF signalling and pro-inflammation is parallel with the increase in adipogenesis, it is interesting to speculate that inflammatory proteins might act as sensors that can exert metabolic regulation on cells.

### Role of stress hormones in metabolic regulation

It appears that co-culturing did not contribute to pre-adipocyte differentiation or PPARγ mediated adipogenesis. This is why we chose to work with stabilized pre-adipocytes over differentiated adipocytes. Co-cultured pre-adipocytes treated with the stress hormones cortisol and serotonin together expressed higher PPARγ than untreated ones accounting for higher lipid accumulation due to cortisol-serotonin treatment. It is clear that cortisol and serotonin act synergistically to induce higher differentiation and adipogenesis in co-cultured pre-adipocytes. An increase in aquaporins AQP7 (Fig. S3) and AQP11 (see Table S1) with time due to cortisol-serotonin treatment most likely indicates that extracellular glycerol content in the co-culture is high and that AQP7 helps to transport the free glycerol into pre-adipocytes, where they can be used for triglyceride synthesis. This suggests that macrophages in the co-culture might be undergoing lipolysis to account for the extracellular glycerol excess. It was observed that the lipid content of macrophages decreased with time due to treatment with cortisol and serotonin (Fig. S3). Thus the hormones cortisol and serotonin act together to mobilize lipids from macrophages through lipolysis, which act as source for FA bio-synthesis in pre-adipocytes. In addition to this the metabolic by-products of AA (PGs, HETE, PCs) are also potential ligands for PPARγ (Ibabe et al., 2005; Nosjean and Boutin, 2002) and might be responsible for the higher lipid content in cortisol-serotonin treated pre-adipocytes.

## MATERIALS AND METHODS

### Cell culture

Murine pre-adipocyte cells 3T3-L1 and murine macrophage cells RAW 264.7, were purchased from the national cell repository at the National Center for Cell Science (NCCS) at Pune, India. 3T3-L1 pre-adipocytes were cultured in Dulbecco's modified Eagle's medium (DMEM, HiMedia, India) supplemented with 10% (v/v) heat inactivated fetal bovine serum (HiMedia), 1 mM L-Glutamine (Sigma-Aldrich) and Amphotericin-B (Sigma-Aldrich) and Gentamycin (Sigma-Aldrich) at 37°C in humidified atmosphere of 95% air and 5% CO_2_. The medium was renewed every 2–3 days and the cells were harvested for sub-culturing by 0.25% trypsin with 1 mM EDTA (Sigma-Aldrich) for 3 min at 37°C when the confluence reached 70%. RAW 264.7 murine macrophage-like cell line was cultured in DMEM supplemented with 10% (v/v) heat inactivated fetal bovine serum (HiMedia), 2% L-Glutamine and Amphotericin-B and Gentamycin under similar atmospheric conditions. The cells were harvested for sub-culturing at 80% confluence.

Indirect (transwell) co-culture was performed by incubating RAW 264.7 cells (1×10^6^ cells) in 0.4 μm-pore-size cell culture inserts (BD Bioscience) and placing them in six-well plates containing 3T3-L1 adipocytes stabilized for 2 days (1×10^6^ cells). In another set-up, 3T3-L1 (3×10^5^ cells) were grown in 0.4 μm-pore-size cell culture inserts, stabilized for 2 days and then placed in six-well plates containing RAW 264.7 (2×10^6^ cells) macrophage cells. In both, the co-culture set-ups were incubated with 100 µM cortisol, 100 µM serotonin and 100 µM cortisol-serotonin for 6 h, 24 h and 48 h. Both cortisol and serotonin were purchased in powder form from MP Biomedicals (Bulingame, USA) and the stock solution was made in DMSO, while the working solution was made by diluting in Mili-Q water (Millipore). Media change was done after 24 h and the new media was also supplemented with 100 µM cortisol, 100 µM serotonin and 100 µM cortisol-serotonin until harvesting was done. Cells were harvested from the lower well for RNA extraction for each setup and supernatant was collected for cytokine estimation.

### Cytokine detection

TNF-α, IL-1β, IL-6, IL10, IL12 and TGFβ in cell culture supernatants were also measured by ELISA using antibodies for anti-TNF alpha (catalogue no. ab6671), anti-IL1 beta (catalogue no. ab9722), anti-IL6 (catalogue no. ab7737), anti-IL10 (catalogue no. ab9969), anti-IL12 (catalogue no. ab7737) and anti-TGF beta1 (catalogue no. ab64715). ELISA plates were coated with supernatant overnight at room temperature. After 1 h of blocking, antibodies were added to each well and were incubated at room temperature for 6 h. Wells were washed three times with PBS supplemented with 0.5% Tween 20 (PBST). Biotin-conjugated detection antibodies were added and incubated at room temperature for 2 h. Alkaline phosphatase-conjugated streptavidin was then added and incubated at room temperature for 1 h. After three washes, the substrate was added to the wells. Within 45 min, the reaction was stopped by the addition of 50 μl of 1N H_2_SO_4_, and absorbance was assessed using a Bio-Rad microplate reader, model 680 at 450 nm.

### Quantitative PCR

Total RNA from RAW 264.7 or 3T3L1 cells was extracted by using RNeasy Mini Kit (Qiagen). cDNA was synthesized from RNA using AffinityScript One-Step RT-PCR Kit (Agilent, Santa Clara, USA) as per the manufacturer's protocol. Briefly, 5 µg of total RNA was mixed with the buffer containing Affinity Script reverse transcriptase and polyT primer. The mixture was kept in the thermo cycler at 45°C for 30 min to synthesize cDNA. Then the temperature was raised to 92°C for 1 min to deactivate the enzyme.

qRT-PCR reaction was set in a 96-well PCR plate. The template, required primer, buffer and SYBR green along with DNA polymerase were added in PCR plate as per the manufacturer's protocol. The plate was then kept in the q-RT-PCR machine (Mx3005P, Stratagene, La Jolla, USA) and the machine was programmed as follows: 2 min at 92°C to activate DNA polymerase for 1 cycle, 15 s at 92°C for melting and 1 min at 60°C for primer annealing along with extension of the chain and detection of the florescence for 40 cycles, then a program to find out the melting temperature of each product. Cycle threshold values were noted and fold changes of the desired genes were calculated with respect to the control after normalizing with internal control gene β-ACTIN. The qRT-PCR reactions were set up as three technical replicates along with no template control and no primer control.

### Microarray

A separate set of 3T3L1 adipocytes were grown (both grown individually as well as co-cultured) and treated with the stress hormones cortisol 100 µM, serotonin 100 µM and cortisol-serotonin 100 µM for 6 h, 24 h and 48 h as described in the cell-culture section of the Materials and Methods. Total RNA was extracted using RNAeasy Kit (Qiagen). The RNA samples for gene expression were labelled using Agilent Quick-Amp labelling Kit (p/n5190-0444). 2000 ng each of the time matched untreated and cortisol, serotonin and cortisol-serotonin treated RNA samples were incubated with reverse transcription mix at 40°C and converted to double stranded cDNA primed by oligodT with a T7 polymerase promoter. Synthesized double stranded cDNA were used as template for cRNA generation. The cDNA synthesis and *in vitro* transcription steps were carried out at 40°C. cRNA was generated by *in vitro* transcription. In the kinetic study, Cy5 CTP dye was incorporated in co-cultured adipocytes cRNA and was Cy3 CTP dye was incorporated in the cRNA of time matched adipocytes grown alone for all the conditions (no treatment, cortisol, serotonin and cortisol-serotonin). In the comparison study for the effect of cortisol, serotonin and cortisol-serotonin on co-cultured adipocytes at 48 h, Cy3 CTP dye was incorporated in the pooled cRNA from 48 h untreated adipocytes and macrophages grown alone, and Cy5 CTP dye was incorporated in the cRNA of co-cultured adipocytes for all conditions. The labelled cRNA samples were hybridized to 4x44k microarray slides. 825 ng each of Cy3 and Cy5 labelled samples were fragmented and hybridized. Fragmentation of labelled cRNA and hybridization were done using the Gene Expression Hybridization kit of Agilent (part number 5188–5242). Hybridization was carried out in Agilent Surehyb Chambers at 65°C for 17 h. The hybridized slides were washed using Agilent Gene Expression wash buffers (part number 5188–5327). Data extraction from the images was done using Agilent Feature Extraction software Version 10.7.

### Data analysis

Feature extracted data were analysed in the online webserver Arraypipe version 2.7. For the kinetics study, significantly differentially regulated genes were calculated directly as normalized signal ratios of co-cultured adipocytes and adipocytes grown alone for each time-matched condition (i.e. no treatment, cortisol, serotonin and cortisol-serotonin) and genes with 1.5-fold changes and above, were identified. For the comparison study for the effect of cortisol, serotonin and cortisol-serotonin on co-cultured adipocytes at 48 h, normalized CY5 to CY3 signal ratio of cortisol, serotonin and cortisol-serotonin were divided by normalized CY5 to CY3 signal ratio of no treatment to calculate the significantly differentially-regulated genes in each hormonal treatment. Differentially regulated genes parsed with InnateDb (www.innatedb.com) and KEGG pathways and Bioconductor in R to find out the pathways populated with the differentially regulated genes. Heat-maps have been used to represent the fold changes of genes in the form of colour coding. Both Excel and R have been used for the generation of heat-maps. Gene Ontology enrichment was done using Panther (www.pantherdb.org).

In the figures, fold changes are expressed as mean±s.d. of the probe replicates for a given gene in the array.

All other statistical analysis was performed using GraphPad Prism version 5.04 software. Statistically significant differences were assessed using two-way ANOVA, followed by Bonferroni post-test for multiple comparisons. All values are presented as the mean±s.d. Statistical significance was assigned at *P*<0.05.

## Supplementary Material

Supplementary information
