# Supplementary material for: Role of murine macrophage in temporal regulation of cortisol- and serotonin-induced adipogenesis in pre-adipocytes when grown together
Source: Biol Open. 2018 Aug 10;7(8):bio034629. doi: 10.1242/bio.034629 (PMC6124570; doi:10.1242/bio.034629)
Supplement: Supplementary information [file biolopen-7-034629-s1.pdf]

Supplementary Materias

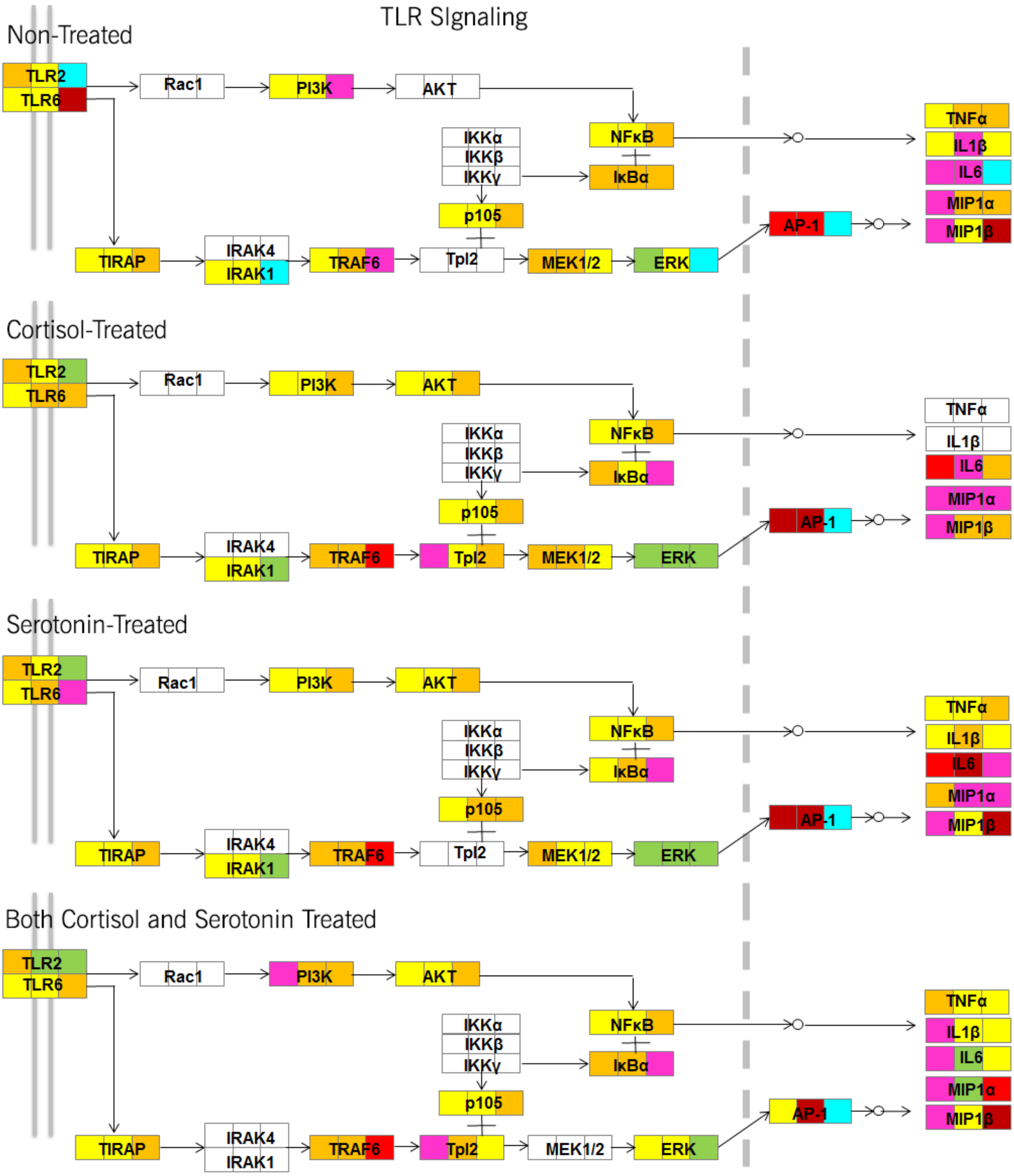

Fig. S1. Time dependent TLR pathway gene expression. Kinetics of gene expression at 6, 24 and 48h in TLR signaling pathway following no treatment and treatment with cortisol,

Biology Open • Supplementary information

serotonin and cortisol and serotonin together is shown with color coding in VIBGYOR mode, where blue indicates down regulation and red denotes up-regulation and yellow represents no or insignificant changes.

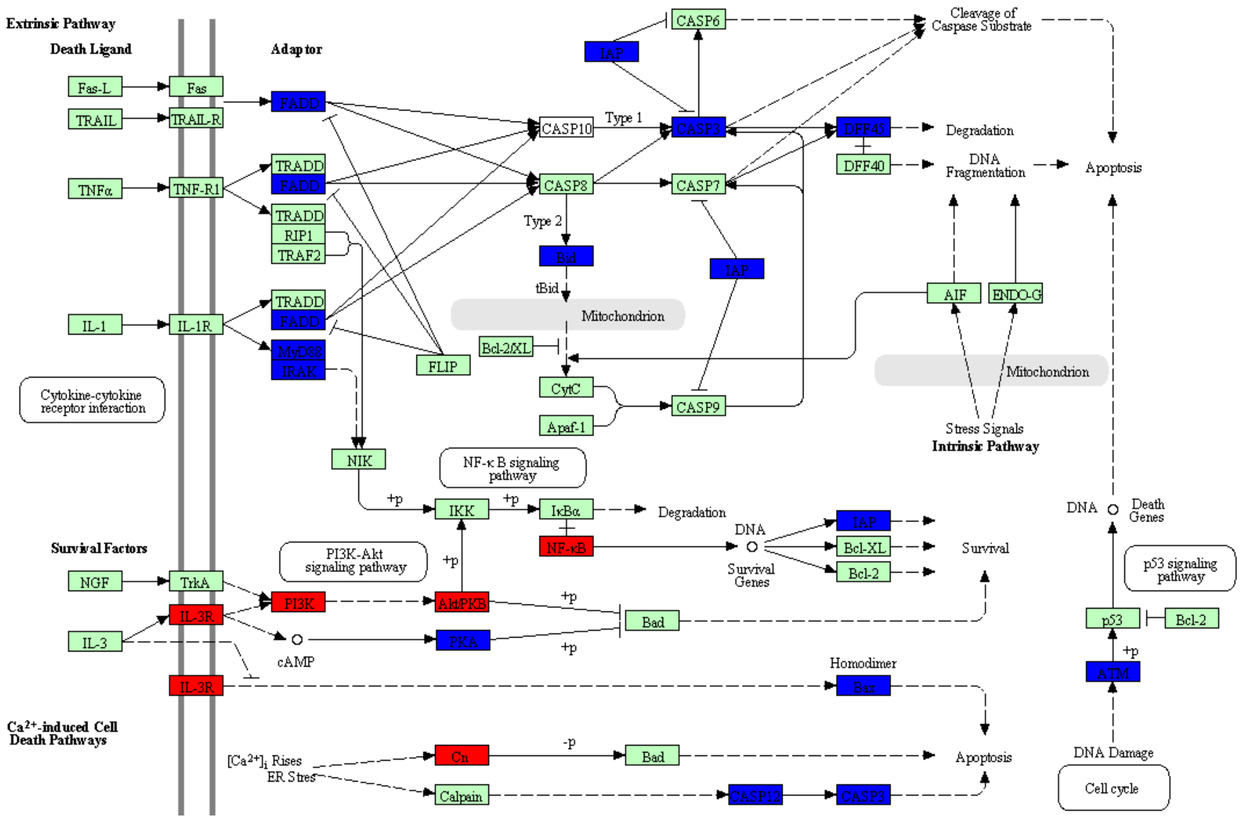

Fig. S2. Time dependent gene expression of Death pathway. Apoptotic pathway following treatment with cortisol at 48h is shown. Data is shown with similar color code as described in Fig.S1. Trend of the data for S and CS treatment were also similar.

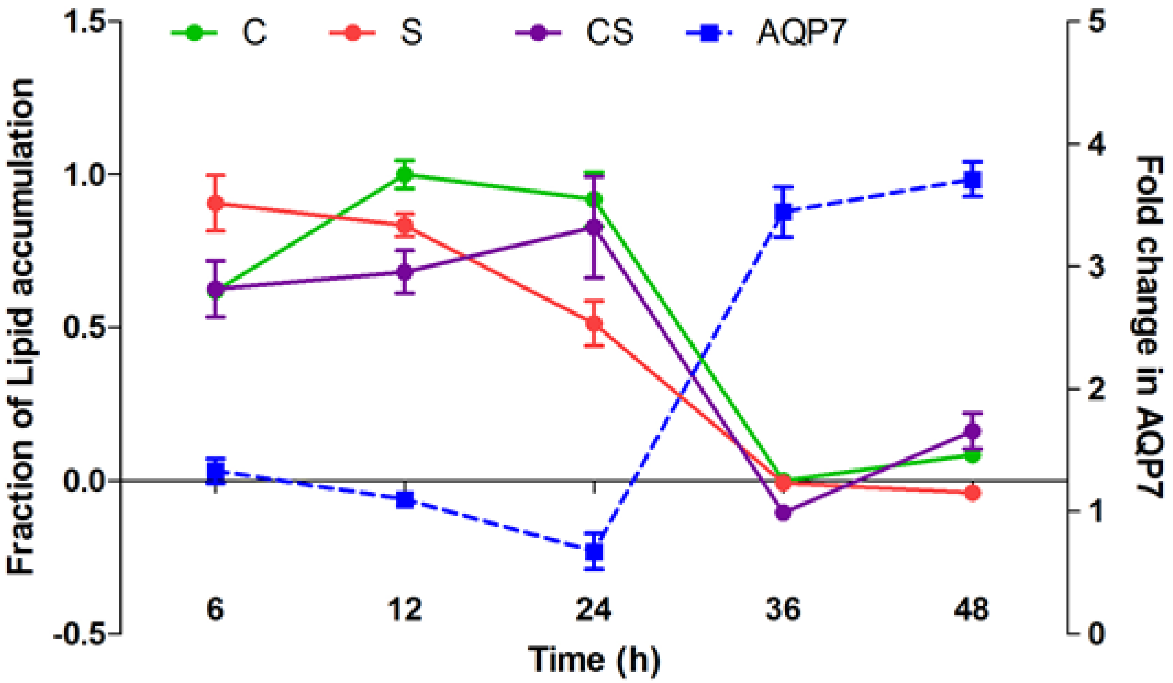

Fig. S3. Lipid accumulation. Kinetics of fraction of lipid accumulation following treatment with cortisol (green line), serotonin (red line) and cortisol and serotonin together (purple line) is shown on left 'Y' axis. Kinetics of fold change of AQP7 (blue line) is shown following treatment with cortisol.

Table S1: Differential transcription values of genes expressed as fold-changes obtained from microarray of co-cultured adipocytes treated with NT, C, S and CS at 6h, 24h and 48h compared against time-matched adipocytes grown alone; fold-change cut off used is +/- 2.

	6NT	6C	6S	6CS	24NT	24C	24S	24CS	48NT	48C	48S	48CS
LOC101055971	1.0	1.0	1.0	1.0	1.0	1.0	1.0	1.0	11.3	15.9	14.7	18.1
Gm4470	1.0	1.0	1.0	1.0	1.0	1.0	2.0	1.0	10.0	5.8	7.0	4.9
A830049F12Rik	1.0	1.0	1.0	1.0	1.0	1.0	1.0	1.0	8.1	6.6	6.1	8.2
Glis1	1.0	1.0	1.0	1.0	1.0	1.0	2.2	1.0	7.1	7.2	5.7	6.8
Gm22	1.0	1.0	1.0	1.0	1.0	1.0	2.2	1.0	6.9	4.3	7.0	4.8
Pvrl2	1.0	1.0	1.0	1.0	1.0	1.0	1.0	1.0	6.8	3.2	3.1	3.6
Lce3c	1.0	1.0	1.0	1.0	1.0	1.0	1.0	1.0	6.5	3.1	5.0	5.6
Galnt6	1.0	1.0	1.0	1.0	1.0	1.0	1.0	1.0	5.7	2.7	3.8	1.0
Grb7	1.0	1.0	1.0	1.0	1.0	1.0	1.0	1.0	5.7	6.4	4.1	6.0
Hist1h2bc	1.0	1.0	1.0	1.0	1.0	1.0	1.0	1.0	5.7	5.6	6.9	4.8
Il4ra	1.0	1.0	1.0	1.0	1.0	1.0	1.0	1.0	5.6	3.5	3.8	3.7
1110002E22Rik	1.0	1.0	1.0	1.0	1.0	1.0	1.0	1.0	5.4	2.6	3.5	4.8
Gm10605	1.0	1.0	1.0	1.0	1.0	1.0	1.0	1.0	5.4	6.2	8.2	7.4
Ost4	1.0	1.0	1.0	1.0	1.0	1.0	1.0	1.0	5.4	2.1	4.0	2.2
Plch2	1.0	1.0	1.0	1.0	1.0	1.0	1.0	1.0	5.3	3.0	3.3	3.2
Gzmm	1.0	1.0	1.0	1.0	1.0	1.0	2.1	2.5	5.2	5.0	5.4	5.2
Cdk5r1	1.0	2.2	1.0	1.0	1.0	1.0	1.0	1.0	5.2	7.9	6.6	9.0
Gm5088	1.0	1.0	1.0	1.0	1.0	1.0	1.0	1.0	5.2	5.7	5.4	5.5
BC100530	1.0	1.0	1.0	1.0	1.0	1.0	1.0	1.0	5.2	8.7	10.3	8.2

Biology Open • Supplementary information

4930421J07Rik	1.0	1.0	1.0	1.0	1.0	1.0	1.0	1.0	5.2	3.0	3.3	3.6
Gm2921	1.0	2.7	1.0	2.3	1.0	1.0	1.0	1.0	5.1	6.7	6.4	6.4
Nfyb	1.0	1.0	1.0	1.0	1.0	1.0	1.0	1.0	5.1	2.5	3.8	3.9
Atp9a	1.0	1.0	1.0	1.0	1.0	1.0	1.0	1.0	5.0	5.4	7.3	7.1
Rab8b	1.0	1.0	1.0	1.0	1.0	1.0	1.0	1.0	4.9	1.0	3.6	2.4
Sik1	1.0	1.0	1.0	1.0	1.0	1.0	1.0	1.0	4.9	5.3	7.1	5.3
Slc23a3	1.0	1.0	1.0	1.0	2.2	1.0	2.2	1.0	4.8	4.1	6.3	5.3
Gjb3	1.0	1.0	1.0	1.0	1.0	1.0	1.0	1.0	4.8	4.6	5.6	5.5
AI606181	1.0	1.0	1.0	1.0	1.0	1.0	1.0	1.0	4.7	3.1	3.9	5.5
Atp8a1	1.0	1.0	1.0	1.0	1.0	1.0	1.0	1.0	4.7	3.8	3.4	3.7
Dmkn	1.0	1.0	1.0	1.0	1.0	1.0	1.0	1.0	4.6	3.8	4.9	6.4
Dok7	1.0	1.0	1.0	1.0	1.0	1.0	1.0	1.0	4.6	3.8	3.3	3.7
Tnpo1	1.0	1.0	1.0	1.0	1.0	1.0	2.7	1.0	4.6	5.1	4.2	3.9
Lat2	1.0	1.0	1.0	1.0	1.0	1.0	1.0	1.0	4.6	1.0	3.6	2.0
4631405J19Rik	1.0	1.0	1.0	1.0	1.0	1.0	1.0	1.0	4.5	3.5	4.0	3.4
Sccpdh	1.0	1.0	1.0	1.0	1.0	1.0	2.0	1.0	4.5	3.0	3.9	3.0
Tbx3	1.0	1.0	1.0	1.0	1.0	1.0	1.0	1.0	4.5	4.0	5.4	4.4
Tmem191c	1.0	1.0	1.0	1.0	1.0	1.0	1.0	1.0	4.4	3.5	5.8	3.8
Mgarp	1.0	1.0	1.0	1.0	1.0	1.0	1.0	1.0	4.4	2.2	3.9	2.3
Slc17a9	1.0	1.0	1.0	1.0	1.0	1.0	1.0	1.0	4.4	5.5	5.0	5.7
Nrn1	1.0	1.0	1.0	1.0	1.0	1.0	1.0	1.0	4.4	3.9	4.9	3.9
Sec24a	1.0	1.0	1.0	1.0	1.0	1.0	1.0	1.0	4.3	3.6	3.8	4.0
Cspg5	1.0	1.0	1.0	1.0	1.0	1.0	2.3	1.0	4.3	5.1	5.8	6.5
Lrrc8d	1.0	2.0	1.0	1.0	1.0	1.0	1.0	1.0	4.3	4.5	4.9	5.2
Tram2	1.0	1.0	1.0	1.0	1.0	1.0	1.0	1.0	4.2	3.4	4.2	3.6
Rpgrip1	1.0	1.0	1.0	1.0	1.0	1.0	1.0	1.0	4.2	3.1	3.2	2.8
Gm8457	1.0	1.0	1.0	2.2	1.0	1.0	1.0	1.0	4.1	3.5	5.1	4.3
Sox9	1.0	1.0	1.0	1.0	1.0	1.0	1.0	1.0	4.0	3.5	3.8	4.0
Rps6ka1	1.0	1.0	1.0	1.0	1.0	1.0	1.0	1.0	3.9	4.1	4.0	4.4
Dnahc6	1.0	1.0	1.0	1.0	1.0	1.0	1.0	1.0	3.8	3.0	3.8	3.4
Id2	1.0	1.0	1.0	1.0	1.0	1.0	1.0	2.4	3.8	4.4	4.4	5.0
2900097C17Rik	1.0	1.0	1.0	1.0	1.0	1.0	1.0	1.0	3.8	3.9	4.4	3.7
A830073O21Rik	1.0	1.0	1.0	1.0	1.0	1.0	1.0	1.0	3.8	2.2	3.2	3.0
Pdk4	1.0	1.0	1.0	1.0	1.0	1.0	1.0	1.0	3.8	2.8	3.1	2.3
Emilin1	1.0	1.0	1.0	1.0	1.0	1.0	1.0	1.0	3.8	4.2	4.4	4.4
Uap1	1.0	2.1	1.0	1.0	1.0	1.0	1.0	1.0	3.7	4.8	3.8	4.1
Rabgef1	1.0	1.0	1.0	1.0	1.0	1.0	1.0	1.0	3.7	3.8	3.0	2.9
Arhgap22	1.0	1.0	1.0	1.0	1.0	1.0	1.0	1.0	3.7	1.0	3.6	3.0
Nmb	1.0	1.0	1.0	1.0	1.0	1.0	2.2	1.0	3.7	4.0	4.2	3.9
Coq10b	1.0	1.0	1.0	1.0	1.0	1.0	1.0	1.0	3.7	2.6	2.9	2.5
Gm14168	1.0	1.0	1.0	1.0	1.0	1.0	1.0	1.0	3.7	2.8	3.1	2.8
Myo15	1.0	1.0	1.0	1.0	1.0	1.0	1.0	1.0	3.6	3.8	4.5	3.3
Maff	1.0	1.0	1.0	1.0	1.0	1.0	1.0	2.3	3.6	4.0	4.4	5.0
Cfp	1.0	1.0	1.0	1.0	1.0	1.0	1.0	1.0	3.6	3.1	3.1	2.9
Mical3	1.0	1.0	1.0	1.0	1.0	1.0	1.0	1.0	3.6	2.9	3.7	2.9
Erp27	1.0	1.0	1.0	1.0	1.0	1.0	1.0	1.0	3.5	2.6	3.2	3.0
6330416G13Rik	1.0	1.0	1.0	1.0	1.0	1.0	1.0	1.0	3.5	3.0	3.5	3.0
Shd	1.0	1.0	1.0	1.0	1.0	1.0	1.0	1.0	3.5	3.5	3.8	3.2
Hist2h3c2	1.0	1.0	1.0	1.0	1.0	1.0	1.0	1.0	3.5	3.6	5.4	4.2
Traf6	1.0	1.0	1.0	1.0	1.0	1.0	1.0	1.0	3.5	3.2	3.3	3.8
Ppapdc1b	1.0	1.0	1.0	1.0	1.0	1.0	1.0	1.0	3.4	1.0	3.0	2.3
Crabp2	1.0	1.0	1.0	1.0	1.0	1.0	1.0	1.0	3.4	3.5	3.5	3.2
Wdr26	1.0	1.0	1.0	1.0	1.0	1.0	1.0	1.0	3.4	3.7	3.4	3.7
BC062258	1.0	1.0	1.0	1.0	1.0	1.0	1.0	1.0	3.4	3.3	3.3	3.1

Kif5a	1.0	1.0	1.0	1.0	1.0	1.0	1.0	1.0	3.4	3.5	3.8	3.4
Napsa	1.0	1.0	1.0	1.0	1.0	1.0	1.0	1.0	3.4	2.9	3.4	3.3
5430431A17Rik	1.0	1.0	1.0	1.0	1.0	1.0	1.0	1.0	3.4	3.4	3.8	3.5
Zbtb1	1.0	1.0	1.0	1.0	1.0	1.0	1.0	1.0	3.3	4.5	3.8	3.8
Soga1	1.0	1.0	1.0	1.0	1.0	1.0	1.0	1.0	3.3	4.6	5.0	3.7
Mreg	1.0	1.0	1.0	1.0	1.0	1.0	2.1	1.0	3.3	2.7	4.4	2.7
Arrb1	1.0	1.0	1.0	1.0	1.0	1.0	1.0	1.0	3.3	6.0	7.6	7.7
1700019N12Rik	1.0	1.0	1.0	1.0	1.0	1.0	1.0	1.0	3.3	4.7	4.6	4.1
Gpd1	1.0	1.0	1.0	1.0	1.0	1.0	1.0	1.0	3.3	2.5	3.3	2.6
Gdf9	1.0	1.0	1.0	1.0	1.0	1.0	1.0	1.0	3.2	2.6	2.9	3.4
Gal3st2	1.0	1.0	1.0	1.0	1.0	1.0	1.0	1.0	3.2	4.2	4.6	4.2
Lrrc31	1.0	1.0	1.0	1.0	1.0	1.0	1.0	1.0	3.2	3.1	3.0	3.3
Fbxw10	1.0	1.0	1.0	1.0	1.0	1.0	1.0	1.0	3.2	3.1	3.0	3.3
Matk	1.0	1.0	1.0	1.0	1.0	1.0	1.0	1.0	3.2	3.0	4.7	3.4
Bin3	1.0	1.0	1.0	1.0	1.0	1.0	1.0	1.0	3.2	2.5	3.6	2.2
Arid3a	1.0	1.0	1.0	1.0	1.0	1.0	1.0	1.0	3.2	4.1	4.4	5.7
Gdap10	1.0	1.0	1.0	1.0	1.0	1.0	1.0	1.0	3.2	3.0	3.7	3.2
Id4	1.0	1.0	1.0	1.0	1.0	1.0	1.0	1.0	3.2	3.7	4.6	4.0
Fosl2	1.0	1.0	1.0	1.0	1.0	1.0	1.0	1.0	3.1	3.9	4.1	3.9
Lce1c	1.0	1.0	1.0	1.0	1.0	1.0	1.0	1.0	3.1	3.1	3.1	2.4
Jhdm1d	1.0	1.0	1.0	1.0	1.0	1.0	1.0	1.0	3.1	3.1	3.0	3.2
Xpo7	1.0	1.0	1.0	1.0	1.0	1.0	1.0	1.0	3.1	4.7	4.3	5.6
Stfa1	1.0	1.0	1.0	1.0	1.0	2.1	1.0	1.0	3.1	3.1	2.9	2.7
Gpi1	1.0	1.0	1.0	1.0	1.0	1.0	1.0	1.0	3.1	3.0	3.3	2.9
Gm10631	1.0	1.0	1.0	1.0	1.0	1.0	1.0	1.0	3.0	3.5	3.2	3.1
Scml4	1.0	1.0	1.0	1.0	1.0	1.0	1.0	1.0	3.0	3.0	3.1	2.6
Tmem184a	1.0	1.0	1.0	1.0	1.0	1.0	1.0	1.0	3.0	3.9	3.7	3.7
Lce1g	1.0	1.0	1.0	1.0	1.0	1.0	1.0	1.0	3.0	4.5	4.4	4.2
5330406M23Rik	1.0	1.0	1.0	1.0	1.0	1.0	1.0	1.0	3.0	3.6	4.0	4.6
Car15	1.0	1.0	1.0	1.0	1.0	1.0	1.0	1.0	2.9	1.0	3.5	2.2
5330426P16Rik	1.0	1.0	1.0	1.0	1.0	1.0	1.0	1.0	2.9	2.0	3.0	2.1
Cebpb	1.0	1.0	1.0	1.0	1.0	1.0	1.0	1.0	2.9	4.4	4.6	4.8
Gm11412	1.0	1.0	1.0	1.0	1.0	1.0	1.0	1.0	2.9	2.1	3.2	2.5
Procr	1.0	1.0	1.0	1.0	1.0	1.0	1.0	1.0	2.9	3.5	3.7	3.5
2310058N22Rik	1.0	1.0	1.0	1.0	1.0	1.0	1.0	1.0	2.9	2.7	3.0	2.4
Acrbp	1.0	1.0	1.0	1.0	1.0	1.0	1.0	1.0	2.9	2.5	2.9	2.6
LOC100861916	1.0	1.0	1.0	1.0	1.0	1.0	1.0	1.0	2.9	2.5	2.8	3.0
Best2	1.0	1.0	1.0	1.0	1.0	1.0	1.0	1.0	2.9	2.8	3.4	3.1
Cnih2	1.0	1.0	1.0	1.0	1.0	1.0	1.0	1.0	2.9	3.9	3.2	3.1
1110017D15Rik	1.0	1.0	1.0	1.0	1.0	1.0	1.0	1.0	2.9	3.0	3.0	2.9
Cdh2	1.0	2.1	1.0	2.0	1.0	1.0	1.0	1.0	2.9	4.5	6.5	5.6
Slc22a13b-ps	1.0	1.0	1.0	1.0	1.0	1.0	1.0	1.0	2.9	2.4	3.3	2.8
Apol8	1.0	1.0	1.0	1.0	1.0	1.0	1.0	1.0	2.8	5.3	5.9	5.4
Plcd4	1.0	1.0	1.0	1.0	1.0	1.0	1.0	1.0	2.8	1.0	3.3	2.6
Dip2b	1.0	1.0	1.0	1.0	1.0	1.0	1.0	1.0	2.8	3.0	2.9	2.9
Vasn	1.0	1.0	1.0	1.0	1.0	1.0	1.0	1.0	2.8	3.5	3.5	3.4
5830469G19Rik	1.0	1.0	1.0	1.0	1.0	1.0	1.0	1.0	2.8	3.3	3.3	2.6
Dnahc10	1.0	1.0	1.0	1.0	1.0	1.0	1.0	1.0	2.8	3.4	4.9	4.0
Evpl	1.0	1.0	1.0	1.0	1.0	1.0	1.0	1.0	2.7	3.5	3.6	3.7
Adssl1	1.0	1.0	1.0	1.0	1.0	1.0	1.0	1.0	2.7	4.2	4.5	4.1
Pde4c	1.0	1.0	1.0	1.0	1.0	1.0	1.0	1.0	2.7	2.8	3.3	3.6
Tspan33	1.0	1.0	1.0	1.0	1.0	1.0	1.0	1.0	2.7	4.1	4.0	5.1
Tubb2a	1.0	1.0	1.0	1.0	1.0	1.0	1.0	2.1	2.7	4.0	3.7	4.3
Mettl6	1.0	1.0	1.0	1.0	1.0	1.0	1.0	1.0	2.7	3.4	3.1	2.5

Biology Open • Supplementary information

Tpsb2	1.0	1.0	1.0	1.0	1.0	1.0	1.0	1.0	2.7	4.1	5.5	4.7
Mansc1	1.0	1.0	1.0	1.0	1.0	1.0	1.0	1.0	2.7	6.2	6.2	5.7
1700109H08Rik	1.0	1.0	1.0	1.0	1.0	1.0	1.0	1.0	2.7	1.0	2.8	2.5
D030002E05Rik	1.0	1.0	1.0	1.0	1.0	1.0	1.0	1.0	2.7	3.1	2.9	3.3
Lmbr1l	1.0	1.0	1.0	1.0	1.0	1.0	1.0	1.0	2.6	2.6	3.1	2.9
Csrnp1	1.0	1.0	1.0	1.0	1.0	1.0	1.0	1.0	2.6	2.8	3.2	3.5
Flcn	1.0	1.0	1.0	1.0	1.0	1.0	1.0	1.0	2.6	3.0	3.0	3.3
Rusc2	1.0	1.0	1.0	1.0	1.0	1.0	1.0	1.0	2.6	3.0	3.1	3.2
Smg7	1.0	1.0	1.0	1.0	1.0	1.0	1.0	1.0	2.6	2.6	3.3	3.6
Khdrbs1	1.0	1.0	1.0	1.0	1.0	1.0	1.0	1.0	2.6	2.5	3.4	2.9
Tigd3	1.0	1.0	1.0	1.0	1.0	1.0	1.0	1.0	2.6	2.5	3.1	2.4
Gramd1c	1.0	1.0	1.0	1.0	1.0	1.0	1.0	1.0	2.6	3.1	2.9	3.6
Tbkbp1	1.0	1.0	1.0	1.0	1.0	1.0	1.0	1.0	2.6	3.3	3.3	3.2
Taf8	1.0	1.0	1.0	1.0	1.0	1.0	1.0	1.0	2.6	4.4	3.1	4.0
Cryab	1.0	1.0	1.0	1.0	1.0	1.0	1.0	1.0	2.5	3.1	3.5	3.4
Gm7092	1.0	1.0	1.0	1.0	1.0	1.0	1.0	1.0	2.5	2.8	2.8	3.0
Inhba	1.0	1.0	1.0	1.0	1.0	1.0	1.0	1.0	2.5	3.4	4.0	3.8
Fubp1	1.0	1.0	1.0	1.0	1.0	1.0	1.0	1.0	2.5	2.5	3.3	3.0
1600020E01Rik	1.0	1.0	1.0	1.0	1.0	1.0	1.0	1.0	2.5	4.4	3.9	4.5
B230303O12Rik	1.0	1.0	1.0	1.0	1.0	1.0	1.0	1.0	2.5	2.4	2.8	3.5
Efnb2	1.0	1.0	1.0	1.0	1.0	1.0	1.0	1.0	2.4	4.8	6.7	4.1
Ifnar1	1.0	1.0	1.0	1.0	1.0	1.0	1.0	1.0	2.4	3.1	3.3	3.4
Chd7	1.0	1.0	1.0	1.0	1.0	1.0	1.0	1.0	2.4	2.6	3.3	3.2
Elavl1	1.0	1.0	1.0	1.0	1.0	1.0	1.0	1.0	2.4	2.5	3.0	3.3
Acp5	1.0	1.0	1.0	1.0	1.0	1.0	1.0	1.0	2.4	3.3	3.5	3.6
1700001J03Rik	1.0	1.0	1.0	1.0	1.0	1.0	1.0	1.0	2.3	2.1	3.3	3.7
Ids	1.0	1.0	1.0	1.0	1.0	1.0	1.0	1.0	2.3	3.0	3.2	2.9
Mslnl	1.0	1.0	1.0	1.0	1.0	1.0	1.0	1.0	2.3	1.0	3.5	1.0
Zbtb32	1.0	1.0	1.0	1.0	1.0	1.0	1.0	1.0	2.3	3.6	3.7	4.0
Fbxo28	1.0	1.0	1.0	1.0	1.0	1.0	1.0	1.0	2.3	2.5	2.9	1.0
Ppfia4	1.0	1.0	1.0	1.0	1.0	1.0	1.0	1.0	2.3	4.5	5.5	5.0
Ovgp1	1.0	1.0	1.0	1.0	1.0	1.0	1.0	1.0	2.3	2.4	2.8	3.0
Cyp4f16	1.0	1.0	1.0	1.0	1.0	1.0	1.0	1.0	2.3	2.3	2.4	2.3
Hbp1	1.0	1.0	1.0	1.0	1.0	1.0	1.0	1.0	2.3	3.6	3.0	2.8
BB166591	1.0	1.0	1.0	1.0	1.0	1.0	1.0	1.0	2.3	3.0	2.9	3.6
Pvrl1	1.0	1.0	1.0	1.0	1.0	1.0	1.0	1.0	2.2	4.3	4.0	3.1
Safb	1.0	1.0	1.0	1.0	1.0	1.0	1.0	1.0	2.2	2.8	3.5	3.6
Elavl3	1.0	1.0	1.0	2.3	1.0	1.0	2.1	1.0	2.2	2.8	3.1	2.5
Gm10094	1.0	1.0	1.0	1.0	1.0	1.0	1.0	1.0	2.2	3.1	4.2	3.4
1700101I11Rik	1.0	1.0	1.0	1.0	1.0	1.0	1.0	1.0	2.2	2.6	3.0	3.6
Vwce	1.0	1.0	1.0	1.0	1.0	1.0	1.0	1.0	2.2	2.8	2.9	2.7
Serpinf2	1.0	1.0	1.0	1.0	1.0	1.0	1.0	1.0	2.2	2.6	3.3	2.1
Fbxo30	1.0	1.0	1.0	1.0	1.0	1.0	1.0	1.0	2.2	2.6	3.0	2.9
Aqp11	1.0	1.0	1.0	1.0	1.0	1.0	1.0	1.0	2.2	3.5	3.1	3.2
Chpf2	1.0	1.0	1.0	1.0	1.0	1.0	1.0	1.0	2.1	2.6	3.7	2.9
Sgms2	1.0	1.0	1.0	1.0	1.0	1.0	1.0	1.0	2.1	2.5	4.1	3.9
Mafk	1.0	1.0	1.0	1.0	1.0	1.0	1.0	1.0	2.1	2.8	2.9	2.6
Stac3	1.0	1.0	1.0	1.0	1.0	1.0	1.0	1.0	2.1	2.6	2.9	3.2
Mef2a	1.0	1.0	1.0	1.0	1.0	1.0	1.0	1.0	2.0	2.9	3.7	2.5

Table S2A

CS C S type

Table S2B

CS C S type

Biology Open • Supplementary information

Tmem192	13.6	9.3	1.2	S-down
Nfatc3	10.1	8.1	1.6	S-down
Olfr791	7.2	6.3	1.4	S-down
Sfn	6.9	4.9	-1.1	S-down
Uqcrc2	6.5	4.4	-1.2	S-down
Fam134c	5.7	5.3	1.2	S-down
Il31ra	5.4	3.7	1.1	S-down
Olfr150	5.4	4.5	-1.0	S-down
Arhgef15	5.2	4.1	-1.0	S-down
Chst13	4.9	4.7	1.0	S-down
Pkd2	4.9	6.1	1.3	S-down
Tcf4	4.9	2.6	1.1	S-down
Irgm2	4.8	4.2	1.1	S-down
Gm10091	4.5	4.6	1.0	S-down
Sdr16c6	4.3	2.5	-1.0	S-down
Mtor	4.1	3.8	1.1	S-down
Gm3669	3.8	3.1	1.1	S-down
Hps4	3.7	4.2	1.3	S-down
Sipa1l1	3.4	2.9	1.6	S-down
Kif2c	3.2	3.7	1.0	S-down
Fbxo34	3.2	2.5	1.5	S-down
Fut7	3.2	2.4	-1.0	S-down
Chst10	3.1	2.4	1.1	S-down
Kera	3.1	2.9	-1.1	S-down
Krt73	3.1	2.0	-1.6	S-down
Mocs3	3.0	2.7	1.0	S-down
1700128E19Rik	2.8	3.0	-1.2	S-down
Plekhh2	2.8	2.7	-1.0	S-down
Mrpl51	2.7	2.8	1.3	S-down
Trcg1	2.7	2.7	-1.5	S-down
Daam2	2.6	2.1	1.2	S-down
Aldob	2.6	2.5	-1.3	S-down
Nid1	2.6	2.5	1.1	S-down
Olfr917	2.5	3.0	1.3	S-down
Setbp1	2.5	2.1	1.2	S-down
5430439M09Rik	2.5	3.0	-1.4	S-down
Yipf1	2.4	2.2	1.2	S-down
Slc7a15	2.4	2.5	-1.0	S-down
Oprd1	2.4	2.6	-1.1	S-down
1110017D15Rik	2.4	2.4	1.1	S-down
Hras1	2.3	2.2	1.0	S-down
Gm10561	2.3	2.5	-1.0	S-down
Zdhhc2	2.3	2.4	1.4	S-down
Efcab9	2.3	2.5	-1.2	S-down
Gm5927	2.3	2.7	2.0	S-down
4932442L08Rik	2.3	2.1	1.0	S-down
Tmem61	2.3	2.7	1.1	S-down
2700005E23Rik	2.3	2.2	-1.1	S-down
Susd4	2.2	2.2	-1.0	S-down
Litaf	2.2	2.3	-1.0	S-down
Med28	2.1	2.0	1.2	S-down
Six3os1	2.1	3.0	1.9	S-down
41338	2.1	2.3	1.2	S-down
Tnfsf18	2.1	2.3	1.3	S-down
Gpr12	2.1	2.1	-1.0	S-down
H2-M10.2	2.1	2.5	1.3	S-down
Mthfd1l	2.0	2.3	1.3	S-down
Dcaf12l2	2.0	2.1	1.4	S-down
Lhfpl2	2.0	2.4	1.1	S-down
1200007C13Rik	2.0	2.2	1.1	S-down
Scfd2	2.2	2.3	8.4	S-up
Olfr582	2.2	2.0	4.0	S-up
Klhl23	1.1	-1.1	3.8	S-up
Wfdc6b	-1.3	-1.2	3.4	S-up
Psmc5	-1.1	1.2	3.3	S-up
Cylc1	-1.4	-2.3	3.3	S-up
ATP8	-1.3	-1.4	3.2	S-up

Mmp28	1.1	5.5	-1.0	C-up
AY761185	-1.0	4.8	1.0	C-up
Tbx20	2.6	3.9	3.0	C-up
Il20rb	3.0	3.9	3.0	C-up
Rheb	2.8	3.8	2.3	C-up
Krt13	2.7	3.5	1.1	C-up
Rab2b	1.2	3.4	1.2	C-up
Mapk4	1.4	3.4	1.6	C-up
Dhrsx	1.2	3.0	1.1	C-up
Rbm3	1.5	2.9	1.5	C-up
Prr14l	-1.0	2.9	-1.0	C-up
Mrpl52	1.0	2.8	1.2	C-up
Oraov1	-1.1	2.7	1.0	C-up
Aqp9	-1.0	2.7	1.0	C-up
Olfr539	-1.1	2.7	-1.0	C-up
Ak8	1.2	2.6	1.2	C-up
Decr1	1.8	2.6	1.4	C-up
Ankrd34b	-1.1	2.6	-1.1	C-up
Gbf1	-2.1	2.6	1.3	C-up
Hfe2	-1.1	2.6	1.1	C-up
Armcx3	1.4	2.6	1.2	C-up
Raver2	1.1	2.6	-1.2	C-up
Olfr458	1.0	2.6	1.1	C-up
Ptprk	-1.1	2.6	-1.0	C-up
Sftpd	-1.0	2.5	-1.0	C-up
Mis18a	-1.1	2.5	1.0	C-up
Nudt13	-1.1	2.5	1.2	C-up
Ccdc149	-1.0	2.5	1.1	C-up
Pmm1	1.1	2.5	-1.1	C-up
1700047E10Rik	1.1	2.5	1.2	C-up
Ace2	-1.0	2.5	1.0	C-up
Uxs1	1.1	2.4	1.1	C-up
Dpyd	1.1	2.4	-1.1	C-up
Smpd5	-1.1	2.4	-1.1	C-up
5730471H19Rik	1.4	2.4	1.3	C-up
Zfp442	-1.0	2.4	1.1	C-up
Epm2aip1	-1.0	2.4	-1.2	C-up
Txndc2	-1.0	2.4	1.0	C-up
Rmi1	1.2	2.4	1.5	C-up
Ppfia4	-1.2	2.4	1.0	C-up
Gm534	-1.1	2.3	-1.3	C-up
Onecut1	1.2	2.3	-1.2	C-up
4930435E12Rik	-1.0	2.3	1.0	C-up
Fam228b	-1.0	2.3	-1.1	C-up
Rnf220	1.2	2.3	1.2	C-up
Osbpl3	-1.2	2.3	1.0	C-up
BC048671	1.2	2.3	1.1	C-up
Daglb	-1.0	2.3	1.4	C-up
Alg14	-1.0	2.3	-1.1	C-up
Rpf1	1.1	2.2	1.1	C-up
Fam173b	1.0	2.2	1.0	C-up
Ebpl	-1.0	2.2	1.0	C-up
Gm5475	-1.2	2.2	-1.0	C-up
Stx6	1.4	2.2	1.1	C-up
Ppm1b	-1.1	2.2	1.0	C-up
Zfp92	-1.1	2.2	1.2	C-up
Ahcy	1.1	2.2	1.2	C-up
4930413E15Rik	1.0	2.1	1.0	C-up
A030012G06Rik	-1.0	2.1	1.0	C-up
Tmem144	1.1	2.1	1.5	C-up
Clca4	-1.0	2.1	1.0	C-up
Catsperd	-1.4	2.1	1.1	C-up
Carhsp1	1.1	2.1	1.1	C-up
Rhoa	1.2	2.1	-1.0	C-up
Zzz3	-1.0	2.1	-1.1	C-up
Adad1	-1.2	2.1	-1.1	C-up
Inmt	-1.1	2.1	-1.1	C-up

Biology Open • Supplementary information

Nlk	-1.3	-1.4	3.0	S-up
Racgap1	1.2	-1.1	3.0	S-up
Sbds	1.1	-1.1	3.0	S-up
Mettl7a3	-1.5	-3.2	2.8	S-up
Zfp653	-1.7	-2.0	2.8	S-up
Fam69c	-1.0	1.1	2.7	S-up
Slc25a26	-1.2	1.0	2.7	S-up
Pnlip	1.7	1.4	2.7	S-up
Slc16a8	1.2	-1.0	2.6	S-up
Reep4	1.1	1.0	2.6	S-up
Gm1082	-1.0	1.0	2.6	S-up
Slc37a4	1.0	-1.2	2.6	S-up
Odf2	-1.0	1.0	2.6	S-up
Nrp	1.4	1.1	2.5	S-up
Yipf3	1.2	-1.1	2.5	S-up
Nalp1b	-3.2	-2.6	2.5	S-up
Sema3c	-1.0	1.1	2.5	S-up
4933415E08Rik	1.1	-1.1	2.5	S-up
Lsm6	1.9	1.1	2.4	S-up
Olfr498	1.4	1.5	2.4	S-up
Sirt2	1.2	1.2	2.4	S-up
Pitx3	-1.1	1.1	2.3	S-up
Lepr	-1.0	1.1	2.3	S-up
Dctn1	-1.1	-1.3	2.3	S-up
Gm13125	1.1	1.2	2.2	S-up
Kdm4b	1.3	1.2	2.2	S-up
Mboat1	1.2	-1.1	2.2	S-up
Zfp189	1.1	-1.0	2.2	S-up
Pdcd7	-1.1	1.2	2.2	S-up
Cblb	1.1	1.2	2.2	S-up
Alpi	1.0	1.1	2.2	S-up
Pdcd1	-1.1	-1.2	2.2	S-up
Fdx1l	-1.0	-1.1	2.1	S-up
Krtap16-1	-1.2	-1.0	2.1	S-up
Gm10037	-1.2	-1.1	2.1	S-up
Cd1d1	1.0	-1.1	2.1	S-up
Dpm1	1.1	1.1	2.0	S-up
Qdpr	-1.2	1.3	2.0	S-up
Fabp12	1.1	1.1	2.0	S-up
Fbxl17	-1.1	-1.1	2.0	S-up
AI132709	1.2	1.0	2.0	S-up
Txndc11	-1.3	2.1	-1.1	C-up
Hist1h1c	1.2	2.1	1.3	C-up
Mpo	1.1	2.1	-1.0	C-up
Olfr691	-1.1	2.1	-1.1	C-up
Pax3	-1.0	2.0	-1.0	C-up

Table S3: Differential transcription values of genes that were uniquely up-regulated or uniquely down-regulated due to treatment with **(CS),** expressed as fold-changes obtained from microarray of co-cultured adipocytes treated C, S or CS at 48h compared against time-matched untreated co-cultured adipocytes as compared against untreated adipocytes after 48h of co-culturing with macrophages. Fold-change cut off used is +/- 2.

Genes	up	Genes	down
Olfr381	3.2	Pbrm1	-8.3
1785C21Rik	3	Htr3a	-3.6
Lypd6b	3	Col19a1	-3.2
Vpreb3	3	Slc16a5	-3.1
Phactr3	3	4930517G19Rik	-3.1
D1379A8Rik	3	Pex7	-3.0
Gm5148	2.9	Slc22a13b-ps	-3.0
Supt6	2.9	Mrpl36	-2.9
Pemt	2.9	C80998	-2.8
Hcn2	2.9	Gm15753	-2.7
Loxl3	2.8	2600006L11Rik	-2.7
Olfr566	2.8	Fgb	-2.6
Gm1576	2.8	Olfr324	-2.6

Tgfb3	2.8	Arhgef26	-2.5
Mettl3	2.8	Dsn1	-2.5
Dcdc2c	2.8	Abca3	-2.5
S1a4	2.7	Mphosph8	-2.4
Zfp352	2.7	Gltscr1l	-2.4
Slc36a2	2.7	B3galt2	-2.4
Man1a	2.7	Fam71a	-2.4
Klk6	2.7	BC039771	-2.4
Mfsd1	2.6	Serpinb3b	-2.4
Zfp111	2.6	Creb3l3	-2.3
Ccdc59	2.6	9530077C14Rik	-2.3
Tmem74b	2.6	A930007D18Rik	-2.3
AU1612	2.6	Cilp2	-2.3
Rbm7	2.5	Ech1	-2.3
AI31464	2.5	Myt1l	-2.3
Cdnf	2.5	Socs1	-2.3
Des	2.5	Prlr	-2.3
Zfp182	2.5	Arid2	-2.3
Prss1	2.4	9230116L04Rik	-2.2
Cldn2	2.4	A430028G04Rik	-2.2
94378K24Rik	2.4	Dpp10	-2.2
493458A3Rik	2.4	Nr1i2	-2.2
Sstr3	2.4	Ubac2	-2.2
22146H18Rik	2.4	Map3k2	-2.2
Dlx1	2.4	Cd300lb	-2.2
Gna15	2.4	Snupn	-2.2
Grm8	2.4	LOC171588	-2.2
493347I5Rik	2.4	Ankzf1	-2.2
Nos3	2.4	Rrp9	-2.2
172I14Rik	2.4	Huwe1	-2.1
Mrps35	2.3	D930032P07Rik	-2.1
Gm13298	2.3	Gdf3	-2.1
Npm1	2.3	Slc10a3	-2.1
Mgat3	2.3	Znrd1	-2.1
Enc1	2.3	Pla2g4c	-2.1
Bicc1	2.3	LOC668306	-2.1
Map4k5	2.3	Gbf1	-2.1
Il1f6	2.3	Fam163a	-2.1
Gm5458	2.3	Icam4	-2.1
LOC1156153	2.3	Gm7903	-2.1
P2rx3	2.3	Serpina7	-2.1
Tdrd12	2.3	Defb7	-2.1
Synm	2.3	Gm15441	-2.0
Vstm5	2.3	Adamts20	-2.0
Olfr1436	2.3	Sipa1l2	-2.0
Tspo	2.3	Ccdc19	-2.0
Schip1	2.3	4930593A02Rik	-2.0
Arl1	2.3	Olfr355	-2.0
Kdsr	2.2	Hsd17b6	-2.0
Eif3c	2.2	Akirin1	-2.0
Micall1	2.2	LOC100504500	-2.0
Mrps17	2.2	Olfr775	-2.0
Gpsm1	2.2	Canx	-2.0
933184L24Rik	2.2		
Gm242	2.2		
Fam222a	2.2		
Adamts9	2.2		
Cox5b	2.2		
493412O13Rik	2.2		
Olfr713	2.2		
Bad	2.2		
Kcna5	2.2		
Scarf1	2.2		
Nfyc	2.2		
Zfp354a	2.2		
1729M2Rik	2.2		
Fam63b	2.2		
Psmb1	2.2		
Chadl	2.2		
Rybp	2.2		

Biology Open • Supplementary information

Galntl6	2.2
Chrnd	2.2
D7Ertd143e	2.2
Mrpl34	2.2
Ccdc92	2.1
4933411G6Rik	2.1
Zfp74	2.1
Ublcp1	2.1
Tmem39a	2.1
Nlrp9c	2.1
Smad2	2.1
Gm111	2.1
Gm5465	2.1
Mlh1	2.1
Nfkbil1	2.1
Ceacam-ps1	2.1
Ccdc24	2.1
Preb	2.1
Cela2a	2.1
Slc6a9	2.1
